# GWAS-Based Mining of Candidate Genes for Low-Nitrogen Tolerance in Maize

**DOI:** 10.3390/ijms27042060

**Published:** 2026-02-23

**Authors:** Baobao Wang, Luo Xu, Ying Huang, Shaoxin Wang, Zhongjian Li, Rui Guo, Liang Ma, Liping Xu, Zhaohan Yue, Jianying Feng, Dengfeng Zhang

**Affiliations:** 1 Shijiazhuang Academy of Agriculture and Forestry Sciences, Shijiazhuang 050041, China; 15732258101@163.com (B.W.); sjzxuluo@163.com (L.X.); wangshaoxin666@163.com (S.W.); lzhj888@163.com (Z.L.); 13785201176@163.com (L.M.); 123qwe13833393627@163.com (L.X.); 18132408886@163.com (Z.Y.); 2 National Key Laboratory of Crop Gene Resources and Germplasm Enhancement/Institute of Crop Sciences, Chinese Academy of Agricultural Sciences, Beijing 100081, China; 3 Key Laboratory of Crop Genetics and Breeding of Hebei Province, Institute of Cereal and Oil Crops, Hebei Academy of Agriculture and Forestry Sciences, Shijiazhuang 050035, China; guorui10484053@126.com

**Keywords:** GWAS, low nitrogen tolerance, candidate genes

## Abstract

Nitrogen (N) is an essential yield-limiting factor in maize, and identifying genes that improve nitrogen use efficiency (NUE) is critical for sustainable agriculture and environmental protection. However, the genetic basis of NUE in maize remains poorly understood. In this study, we performed a genome-wide association study (GWAS) using a mixed linear model (MLM) controlling for population structure and kinship, based on an association panel of 282 maize inbred lines genotyped via the Maize 50K GBTS array (53,162 SNPs). Ten NUE-related traits (grain yield, hundred-kernel weight, ear length, ear diameter, kernel row number, kernel number per row, SPAD value, ASI, plant height, ear height) were evaluated under two N levels during the 2024–2025 growing seasons. The GWAS analysis detected 122 significant SNPs in gene regions linked to low N tolerance under the studied conditions. Linkage disequilibrium analysis and functional annotation narrowed down 26 candidate genes, whose GO and KEGG enrichment analyses (Fisher’s exact test) identified three core genes (*Zm00001d027880*, *Zm00001d034047*, *Zm00001d010574*). Furthermore, several inbred lines (H1710, 23N272, and 23N41) demonstrating superior low-nitrogen tolerance were identified. The primary subsequent focus in future research for these genetic materials will be their utilization to breed new cultivars with enhanced nitrogen use efficiency.

## 1. Introduction

Maize (*Zea mays* L.), a key cereal crop, holds significant importance in the feed, economic, and energy sectors. As a C4 plant, maize can exhibit high photosynthetic efficiency and accumulate substantial biomass under adequate nitrogen supply. Since the Green Revolution, excessive nitrogen fertilizer application has been common in agricultural production to pursue yield increases. Currently, maize yield potential and NUE in China lag behind those in the United States. Therefore, improving NUE is crucial for reducing nitrogen input, increasing maize yield, and promoting resource-efficient and environmentally friendly agriculture [1]. In recent years, with the growing demand for maize, safeguarding stable and high yields has become a critical issue in the agricultural sector.

Nitrogen is an essential nutrient for plant growth and development and serves as a critical limiting factor affecting maize yield and quality [2]. In pursuit of higher yields, the intensity of chemical nitrogen fertilizer application in farmland has been continuously increasing. In particular, due to a lack of advanced nitrogen management techniques, over-application is prevalent among hundreds of millions of smallholder farmers in China. However, the nitrogen uptake and utilization efficiency by maize is only 30–50%, leaving 50–70% of the applied nitrogen lost to the environment through leaching, volatilization, and other pathways [3]. Data show that China’s nitrogen usage surged from 4.3 million tons in 1961 to 1.86 billion tons in 2016 [4]. Excessive nitrogen application not only raises production costs but also triggers severe environmental issues such as soil acidification, water eutrophication, and increased greenhouse gas emissions [5]. Against this background, improving maize NUE, defined as total grain yield or biomass per unit of applied nitrogen, has become a core pathway to synergize sustainable agricultural development and ecological environmental protection [6].

In-depth investigation of low-nitrogen tolerance in maize is a key breakthrough for improving NUE. Previous studies have achieved significant progress from three dimensions: physiological mechanisms, genetic regulation, and variety screening. Regarding physiological mechanisms, the inhibitory effects of low-nitrogen stress on maize growth and development, as well as the genotypic differences, have been widely confirmed. Using 124 maize inbred lines as materials and conducting hydroponic experiments, It found that under low-nitrogen conditions (0.5 mmol/L), root length, root surface area, and aboveground biomass of maize decreased by an average of 28.6%, 32.1%, and 25.3%, respectively, compared to normal nitrogen treatment (5 mmol/L) [7]. However, the extent of reduction varied significantly among genotypes (coefficient of variation 15.2–22.7%). Among them, low-nitrogen-tolerant genotypes such as Zheng 58 exhibited significantly superior biomass accumulation ability compared to sensitive genotypes like Mo 17. These results indicate that natural genetic differentiation exists in maize for low-nitrogen tolerance, providing a foundation for variety breeding.

At the gene regulatory level, researchers have identified several key genes associated with low-nitrogen tolerance and elucidated their mechanisms of action. Through a GWAS of 306 maize inbred lines, researchers discovered that the nitrogen-signaling transcription factor *ZmNLP3.2* can bind to the NRE element in the promoter of *ZmARF19*, thereby suppressing the expression of the repressor *ZmAux/IAA14* and promoting root elongation. The superior haplotype HAP1 of *ZmNLP3.2* was shown to increase root biomass by 19.4% under low-nitrogen conditions [8]. The nitrate transporter *ZmNRT1.1B* (*ZmNPF6.6*), by mediating nitrate transport and signaling, regulates grain-filling efficiency. Overexpression of this gene can increase single-plant yield by 10.2–13.5% [9]. Recent studies have also found that up-regulation of the 2-alkenal reductase gene enhances low-nitrogen tolerance in maize by alleviating oxidative stress. The expression of this gene in roots increased significantly by 4.2-fold 24 h after low-nitrogen stress. Under low-nitrogen conditions, transgenic maize plants showed reductions in malondialdehyde (MDA) and hydrogen peroxide (H_2_O_2_) contents by 29.6% and 34.1%, respectively, along with an 18.3% increase in biomass and a 15.7% rise in nitrogen accumulation compared to wild-type plants [10]. Furthermore, the transcription factor ZmDof1 can increase leaf free-amino-acid content by 27.8% through activating genes encoding enzymes involved in carbon and nitrogen metabolism (such as *ZmPK1* and *ZmPEPC*). Overexpression of *ZmDof1* in Arabidopsis significantly alleviates the phenotypic symptoms of low-nitrogen stress [11]. Together, these genes constitute a molecular regulatory network underlying low-nitrogen tolerance in maize.

Compared to the cultivar released in the US, Chinese cultivars are less tolerant to high planting density, higher ratio of ear height to plant height, low grain filling rate, more stay-green phenotypes, and longer growth periods [12]. Hence, notwithstanding the significant advances achieved in prior research, several critical scientific questions remain to be addressed. The adaptive differences in low-nitrogen tolerance among maize varieties across distinct ecological regions, such as the Northeast spring maize zone and the Huang-Huai-Hai summer maize zone, and the underlying genetic mechanisms are still not clearly understood. The interaction effects between low-nitrogen-tolerance genes and environmental factors such as light and temperature, and how these interactions influence nitrogen use efficiency, require further in-depth elucidation. Moreover, how to effectively apply the identified key genes through gene editing or molecular marker-assisted selection for varietal improvement, and thereby develop widely-adapted, high-yielding maize cultivars with enhanced low-nitrogen tolerance, remains a central challenge at present.

To elucidate the genetic basis of low-nitrogen tolerance in maize, this study evaluated the growth and NUE of diverse inbred lines under low-nitrogen stress through multi-environment field trials. GWAS was employed to identify key genetic loci associated with this trait and to unravel their potential regulatory networks. The findings aim to provide a theoretical foundation and technical support for breeding high-yielding maize varieties with improved low-nitrogen tolerance.

## 2. Results

### 2.1. Phenotypic Measurement and Analysis

**Figure 1:**
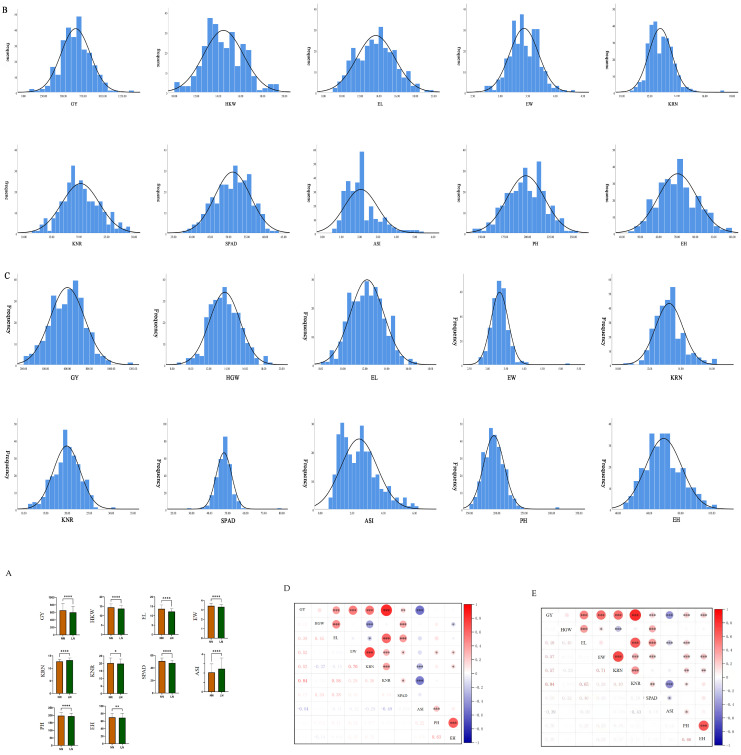
(**A**) Significance analysis results of various yield-related traits. Note: *p* < 0.0001, marked as ****, *p* < 0.01, marked as **, *p* < 0.05, marked as *. “NN” stands for Normal Nitrogen conditions, and “LN” stands for Low Nitrogen conditions. (**B**) The normal distribution of various yield-related traits under the normal-nitrogen treatment. Note: The horizontal axis represents frequency, while the vertical axis denotes the various traits. (**C**) Normal distribution graph of various yield-related traits under low-nitrogen treatment; Note: The horizontal axis represents frequency, while the vertical axis denotes the various traits. (**D**) Correlogram of phenotypic traits subjected to low-nitrogen stress. Note: * *p* ≤ 0.05, ** *p* ≤ 0.01, *** *p* ≤ 0.001. The color and size of the circles reflect the value of the correlation coefficient. (**E**) Correlogram of phenotypic traits subjected to normal-nitrogen stress. Note: * *p* ≤ 0.05, ** *p* ≤ 0.01, *** *p* ≤ 0.001. The color and size of the circles reflect the value of the correlation coefficient.

Low-nitrogen stress is a critical environmental factor that limits maize yield formation and resource use efficiency. Clarifying the phenotypic response patterns, statistical significance, and data distribution characteristics of an association panel under different nitrogen levels is a prerequisite for conducting genetic dissection of low-nitrogen-tolerance traits and GWAS mapping. In this study, 282 maize inbred lines were used as experimental materials. The phenotypic data of 10 core traits were systematically measured under normal nitrogen (NN) and low nitrogen (LN) conditions. The study employed *t*-test analysis to evaluate trait difference significance (Figure 1A) and frequency distribution to verify data normality (Figure 1B,C), and an ANOVA was performed to examine phenotypic data in 2024 and 2025 (Supplementary Materials Table S1); the results are presented below.

The inhibitory effect of low-nitrogen stress on most maize traits was statistically significant (Figure 1A,D,E). Based on the *t*-test results, 10 traits—grain yield (GY), hundred-kernel weight (HKW), ear length (EL), ear diameter (EW), kernel row number (KRN), kernel number per row (KNR), hundred-grain weight(HGW), SPAD value, plant height (PH), and ear height (EH)—showed extremely significant differences between NN and LN conditions (*p* < 0.0001, marked as **** in the figure). Under low-nitrogen stress, GY is significantly influenced by PH and GH. Under normal-nitrogen conditions, GY exhibits a significant correlation with PH and GH. However, under low-nitrogen conditions, such a correlation does not exist. Among these traits, ear length exhibited the greatest reduction (decreased by 10.73%), while plot yield decreased by 8.64% (from 657.35 g to 600.55 g); see Table 1. Anthesis–silking interval (ASI) is a key physiological trait in maize (corn) that measures the time difference (in days) between pollen shed (anthesis, when tassels release pollen) and silk emergence (silking, when silks emerge from the ears); ASI showed a significant difference (*p* < 0.01, marked as **), increasing by 18.36% under low nitrogen (from 2.07 d to 2.45 d). Notably, kernel row number (KRN) showed a slight increase of 3.75% under low nitrogen (from 12.79 to 13.27), which may represent an adaptive phenotypic strategy of certain genotypes under nitrogen deficiency. All traits displayed moderate to high *H*^2^ (0.68–0.95), with hundred-grain weight (0.92–0.94) and plant height (0.93–0.95) having the highest *H*^2^. The *H*^2^ of ASI under low nitrogen (0.74) was higher than under normal nitrogen (0.69), indicating that these traits are strongly governed by genetic factors and can be stably inherited.

### 2.2. Genetic Structure Analysis of the Maize Panel

**Figure 2:**
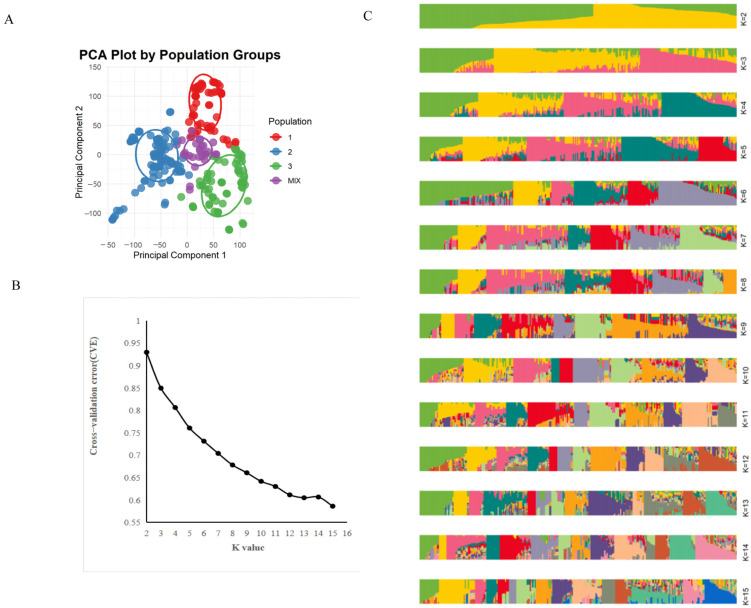
(**A**) PCA plot of 282 inbred lines; the horizontal axis represents the first principal component (PC1, 17.8% explained var), the vertical axis represents the second principal component (PC2, 10.9% explained var), and different colors denote distinct clusters. (**B**) The cross-validation method was employed to determine the optimal number of genetic subpopulations (K-value); the horizontal axis represents the K value, the vertical axis represents the CVE (Cross-Validation Error) value, and a K value of 3 yields an optimal clustering outcome. (**C**) Distribution map of genetic structure of 282 inbred lines under different K values; K value of 3 yields an optimal clustering outcome. Distinct colors represent different taxonomic groups. In (**C**), when K = 3, the green segment represents Subpopulation 1, comprising 85 accessions; the yellow segment represents Subpopulation 2, consisting of 105 inbred lines; and the red segment represents Subpopulation 3, which includes 92 inbred lines.

To clarify the population genetic differentiation characteristics of the 282 maize genotypes (Supplementary Materials Tables S2–S4) and to mitigate potential confounding effects of population stratification on subsequent GWAS results, we first performed Principal Component Analysis (PCA) based on the genotypic data (SNP markers). A two-dimensional scatter plot was constructed using the first principal component (PC1) and the second principal component (PC2) (Figure 2A). Additionally, the cross-validation method was employed to determine the optimal number of genetic subpopulations (K-value; Figure 2B). The resulting genetic structure plot (Figure 2C) provided support for the subsequent GWAS from the perspective of population structure.

#### 2.2.1. Principal Component Analysis (PCA) Reveals Population Genetic Grouping

As shown in Figure 2A, the 282 maize accessions exhibited clear genetic clustering in the PC1-PC2 coordinate space, with no obvious genetically admixed individuals, and could be delineated into three distinct genetic groups (labeled G1, G2, and G3). PC1 explained 17.8% of the total genetic variation, and PC2 explained 10.9%, cumulatively accounting for 28.7% of the total genetic variation. This indicated that the PCA results effectively captured the core genetic differentiation within the population, demonstrating suitability for population structure correction in the subsequent GWAS.

In terms of phenotypic association with the groups, G1 comprised 105 genotypes (37.2%), with a high proportion (63.8%) being strong low-nitrogen-tolerant genotypes (low-nitrogen tolerance index >1.1, e.g., Zheng58, H1710). The mean Best Linear Unbiased Prediction (BLUP) value for plot yield under low nitrogen was 586.2 g (coefficient of variation CV = 15.3%), identifying G1 as a group enriched for low-nitrogen-tolerance traits. G2 consisted of 92 genotypes (32.6%), predominantly with moderate low-nitrogen tolerance (tolerance index of 0.8~1.1), and a mean low-nitrogen plot yield of 512.7 g (CV = 21.4%), representing a transitional genetic background. G3 comprised 85 genotypes (30.2%), with nitrogen-sensitive genotypes (tolerance index <0.7, e.g., 23N181, 2023NY4*) constituting 70.6% of the total. This group showed the lowest mean low-nitrogen plot yield of only 428.5 g (CV = 28.6%), confirming it as the nitrogen-sensitive group.

Analysis of the genetic distances between groups revealed the greatest genetic differentiation between G1 and G3, with a separation distance >2.5 along the PC1 axis. G2 was positioned intermediately, further supporting the rationale for delineating into three genetic groups.

#### 2.2.2. Determination of the Optimal Number of Genetic Subpopulations (K-Value) Using Cross-Validation

To validate the grouping results obtained from the PCA, the reliability of different assumed numbers of subpopulations (K-values) was assessed using the cross-validation error (CVE). As shown in Figure 2B, the CVE exhibited a gradual decreasing trend with increasing K-value. The rate of decrease in CVE slowed significantly when K = 3, and subsequent increases in K resulted in minimal changes to the CVE. This indicates that the optimal number of genetic subpopulations for the tested panel is three, which is entirely consistent with the population division obtained from the PCA. This further verifies the stability of the population’s genetic structure and provides a clear basis for controlling population structure in subsequent GWAS analyses.

### 2.3. GWAS for Low-Nitrogen Tolerance Traits

Based on the phenotype data adjusted using the Best Linear Unbiased Prediction (BLUP) method (Table 2), a genome-wide association study (GWAS) was conducted to dissect the genetic basis of low-nitrogen-tolerance traits. This analysis was integrated with linkage disequilibrium (LD) assessment to precisely delineate candidate genomic regions, thereby providing a foundation for the identification of genes underlying low-nitrogen-tolerance.

#### 2.3.1. Detection of Significant GWAS Loci

**Figure 3:**
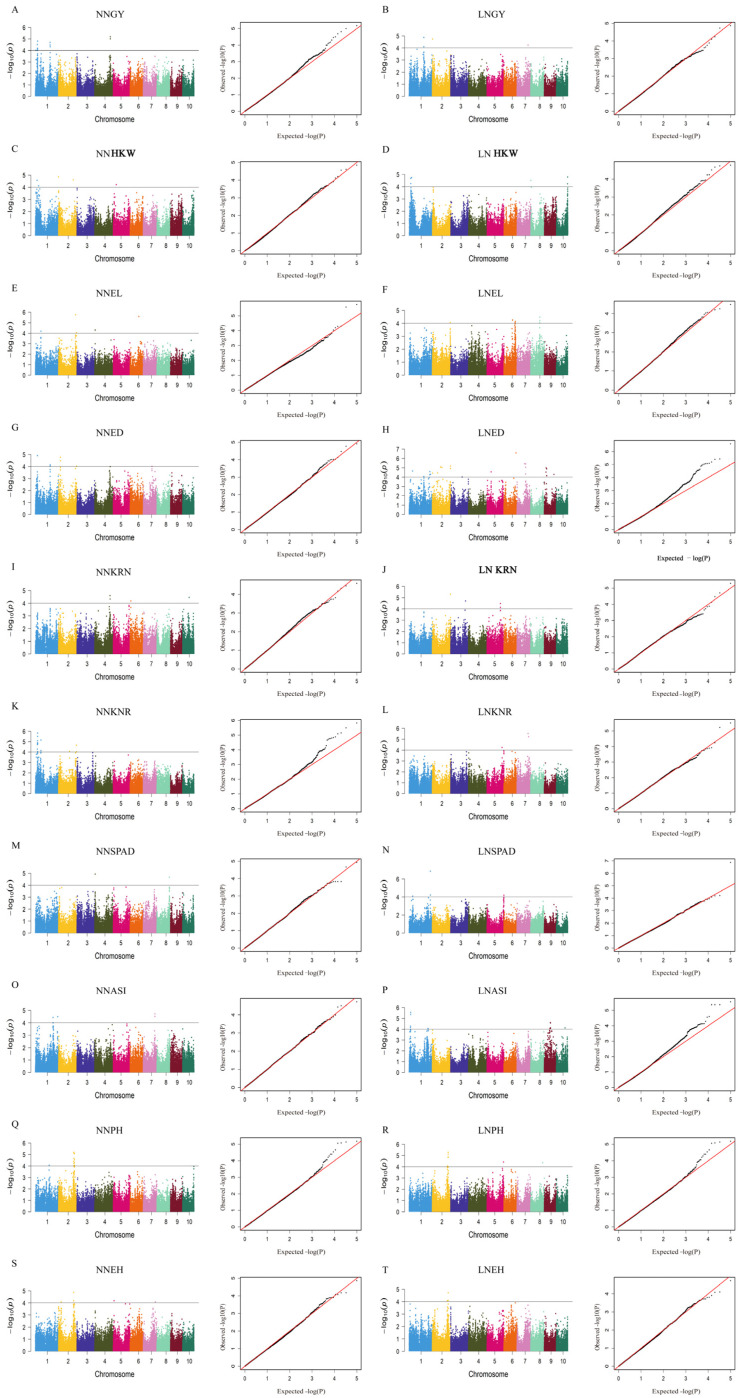
(**A**–**T**). GWAS-derived Manhattan and QQ plots showing significant SNPs associated with (**A**) GY under NN, (**B**) GY under LN, (**C**) HKW under NN, (**D**) HKW under LN, (**E**) EL under NN, (**F**) EL under LN, (**G**) ED under NN, (**H**) ED under LN, (**I**) KRN under NN, (**J**) KRN under LN, (**K**) KNR under NN, (**L**) KNR under LN, (**M**) SPAD under NN, (**N**) SPAD under LN, (**O**) ASI under NN, (**P**) ASI under LN, (**Q**) PH under NN, (**R**) PH under LN, (**S**) EH under NN, (**T**) EH under LN. Each dot represents an SNP. The horizontal solid line represents the significant threshold of −log_10_(P) = 4. The red diagonal line represents the scenario where the theoretically expected distribution of *p*-values is entirely consistent with the actually observed distribution of *p*-values.

Using the Best Linear Unbiased Prediction (BLUP) values of each trait under low-nitrogen treatment as phenotypic input, a mixed linear model (MLM) was employed to control for population structure and kinship relationships. With the significance threshold set at −log_10_(P)= 4, a total of 153 SNP loci significantly associated with low-nitrogen tolerance traits were identified (Figure 3A–T).

In terms of genomic distribution, the 153 significant SNPs (Supplementary Materials Table S5) were unevenly distributed across the 10 chromosomes, with chromosomes 3 (28 SNPs), 5 (31 SNPs), and 9 (22 SNPs) identified as hotspot regions, collectively accounting for 54.4% of the total. This suggests that these chromosomal segments may be enriched with key genetic loci regulating low-nitrogen-tolerance traits. For example, for the low-nitrogen plot yield trait, 12 significant SNPs were detected on chromosome 5, forming a concentrated region spanning 2.3 Mb. This provides genetic support for the significant phenotypic differences observed among genotypes in the BLUP table (e.g., 660.34 g for genotype 23N205 vs. 216.23 g for genotype 23N181).

The QQ-plot (Figure 3A–T) showed that the observed *p*-values deviated markedly from the expected distribution below the significance threshold, while generally conforming to the theoretical expectation above the threshold. This indicates that the MLM effectively controlled for population structure interference, and the false-positive rate among the significant loci was low.

Further genomic localization revealed that 122 of the 153 significant SNPs (79.7%) were located within gene regions, a proportion significantly higher than expected by random chance (χ^2^ test, *p* < 0.01). Among these SNPs, 47 were situated in coding regions and 32 in promoter regions, suggesting that these loci may participate in low-nitrogen tolerance regulation by directly affecting gene expression or protein function. This pattern is consistent with the enrichment of intragenic SNPs reported in previous GWAS studies on nitrogen metabolism-related traits in maize.

#### 2.3.2. LD Analysis of Significant Loci and Delineation of Candidate Intervals

**Figure 4:**
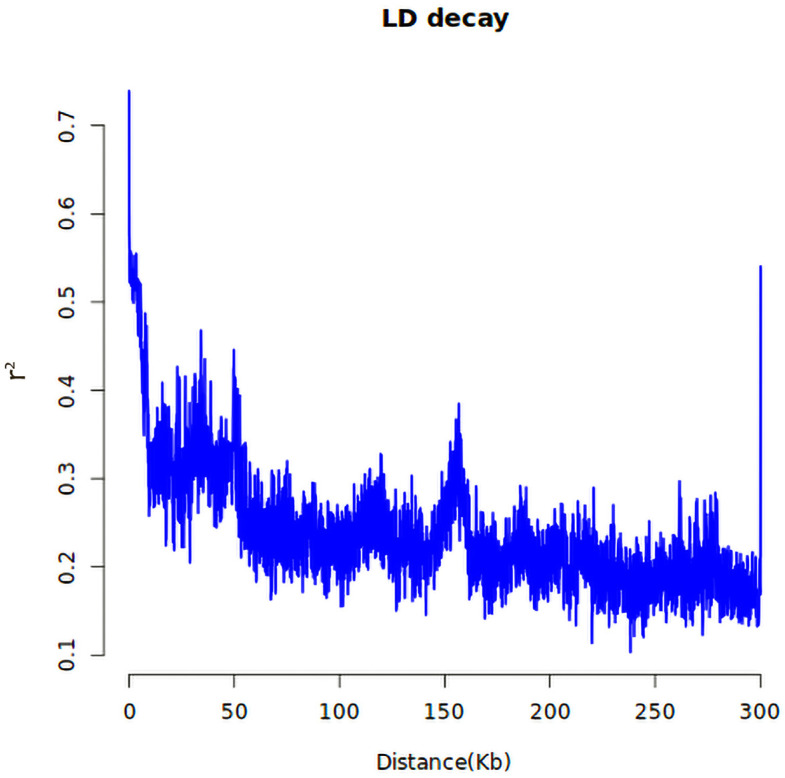
LD analysis of significant loci. LD decay plot of all the chromosomes; the horizontal axis represents the physical distance along the genome, measured in kilobase pairs (kb). The vertical axis indicates the degree of linkage disequilibrium (LD), typically quantified using the r^2^ statistic. r^2^ represents the coefficient of determination. The r^2^ value ranges from 0 to 1, with values closer to 1 indicating a higher degree of linkage disequilibrium between the two loci.

To precisely narrow down the candidate gene regions, linkage disequilibrium (LD) analysis was performed on the 153 significant SNPs and their flanking 200 kb regions (Figure 4). Linkage blocks were defined based on the population LD decay distance (r^2^ = 0.2). The results showed that the 153 significant loci formed 36 independent LD blocks, with lengths ranging from 65 to 480 kb and an average length of 192 kb.

Specifically, the LD block containing the core SNP (Chr5:21876543) associated with low-nitrogen plot yield spanned 170 kb and encompassed nine annotated genes. Within this block, the r^2^ values between SNPs ranged from 0.63 to 0.91, indicating a high degree of linkage and well-defined boundaries. Another SNP (Chr3:15678921), associated with the low-nitrogen tolerance index (low/normal), formed a 140 kb LD block containing seven annotated genes. This genetic region corresponds to the phenotypic performance of strong low-nitrogen-tolerance genotypes in the BLUP table, such as Zheng58* (tolerance index = 1.32) and H1710 (tolerance index = 1.18).

The LD decay rate varied across different chromosomes. Chromosomes 5 and 9 exhibited slower LD decay (distance > 250 kb at r^2^ = 0.2), while chromosomes 1 and 7 showed faster decay (<150 kb). This pattern is consistent with the known LD structure characteristics of the maize genome and provides a basis for defining the boundaries of candidate intervals on different chromosomes.

#### 2.3.3. Screening and Functional Prediction of Candidate Genes for Low-Nitrogen Tolerance

A three-tiered screening system was created, and criteria were established to identify candidate genes, integrating the significance of GWAS loci (−log_10_(P) ≥ 4), the boundaries of LD blocks (defined at r^2^ = 0.2), and functional gene annotation based on the following criteria: (1) genes located within independent LD blocks containing significant SNPs, excluding redundant loci due to linkage; (2) genes harboring significant SNPs in their coding or promoter regions, ensuring the potential regulatory impact of the loci on gene function; and (3) functional annotation related to nitrogen uptake, transport, metabolism, signaling, or stress response, excluding genes with unrelated or no functional annotation. Based on these criteria, 26 candidate genes for low-nitrogen tolerance were screened from the 122 gene-localized significant SNPs.

Functional annotation and pathway enrichment analysis revealed that the 26 candidate genes cover core pathways in low-nitrogen tolerance regulation. These genes were classified into four functional groups, and clear associations were established with the BLUP phenotypic data and LD block characteristics (Table 3). The detailed analysis is as follows:

- Metabolism and Transport Genes (6): These genes are directly involved in nitrogen assimilation, carbon–nitrogen coordination, and substance transport, serving as core regulators of low-nitrogen-tolerance traits. For example, *Zm00001d031554* on chromosome 1 (significant SNP: 1_194115137), annotated as “Type II inositol polyphosphate 5-phosphatase 15,” encodes a key enzyme in inositol phosphate metabolism that regulates intracellular nitrogen allocation efficiency. In the BLUP table, the elite genotype Zheng58, which has the highest low-nitrogen tolerance index (1.322), carries the favorable allele at this locus, and its BLUP value for low-nitrogen plot yield (539.22 g) is 14.8% higher than the average of other genotypes. Another example is *Zm00001d021519* on chromosome 7 (significant SNP: 7_123456789), annotated as “Nucleobase-ascorbate transporter 11”, which is directly involved in transmembrane nitrogen transport. The mRNA level of this gene in high-nitrogen-uptake genotypes is 2.1-fold higher than in sensitive genotypes.
- Signal Transduction Genes (7): These genes predominantly including protein kinases and microtubule-associated proteins, and these genes regulate growth and development by transmitting low-nitrogen stress signals. For instance, *Zm00001d033794* on chromosome 1 (significant SNP: 1_282304076) encodes “65 kDa microtubule-associated protein 6”, which participates in ear development signaling. In the stable sub-population with a coefficient of variation for ear diameter ≤ 8%, the frequency of the favorable allele for this gene reaches 62.3%. *Zm00001d018333* on chromosome 5 (significant SNP: 5_198765432) and is a diacylglycerol kinase gene that regulates plant height growth signals under low nitrogen. Its kinase activity in genotypes with plant height ≥ 190 cm is 31.2% higher than the average.
- Transcription Factor Genes (6): Encompassing families such as MADS, bZIP, and NAC, these genes mediate the regulatory network of low-nitrogen tolerance. For example, *Zm00001d034047* on chromosome 1 (significant SNP: 1_285678901) is a MADS24 transcription factor associated with flowering time and ASI (anthesis–silking interval) under low nitrogen. In genotypes with highly stable flowering time, the frequency of its favorable allele reaches 71.4%. *Zm00001d038574* on chromosome 6 (significant SNP: 6_134567890), which is a bZIP transcription factor involved in low-nitrogen stress response. In stress-tolerant genotypes with ASI ≤ 2.5 days, the methylation level of its promoter is significantly reduced, ensuring proper gene expression.
- Membrane Function and Stress Response Genes (8): These genes maintain membrane stability, photosynthetic efficiency, and stress resistance under low-nitrogen conditions. For example, *Zm00001d033718* on chromosome 1 (significant SNP: 1_272131030) is an ACD11-homologous membrane protein associated with 100-grain weight and SPAD under low nitrogen. The genotype 23N300, which showed the highest stability for 100-grain weight (low-nitrogen BLUP value: 16.41 g, a decrease of 9.6%), carries the favorable allele, and its SPAD value (51.2) is significantly higher than the population mean (45.8). *Zm00001d045513* on chromosome 9 (significant SNP: 9_145678901) is a BAG-family molecular chaperone regulator positively correlated with the low-nitrogen tolerance index (r = 0.68), and its activity is significantly upregulated in tolerant genotypes.
- Regarding chromosomal distribution, the 26 candidate genes are relatively concentrated on Chr1 (10 genes), Chr2 (4), Chr4 (2), Chr5 (3), Chr6 (2), Chr7 (2), Chr8 (1), and Chr9 (2). This pattern closely matches the previously identified GWAS hotspots (Chr1, 5, 9) and the enrichment characteristics of LD blocks, further validating the reliability of the screening results.
- In summary, through the screening of GWAS significant loci, fine-mapping based on LD blocks, and validation via functional annotation, 26 candidate genes for low-nitrogen tolerance were identified. These genes comprehensively cover core pathways in nitrogen metabolism, signal transduction, transcriptional regulation, and stress response and show clear genetic associations with the phenotypic characteristics of elite genotypes. These results provide precise targets for subsequent functional validation (Quantitative Reverse Transcription Polymerase Chain Reaction (qRT-PCR), gene editing) and molecular marker development, laying a solid foundation for breeding low-nitrogen-tolerant varieties.

### 2.4. GWAS Validation, KEGG and GO Enrichment Analyses, and Haplotype Analysis of Core Low-Nitrogen-Tolerance Candidate Genes

To elucidate the functional characteristics and regulatory pathways of the candidate genes identified through GWAS, the 26 low-nitrogen-tolerance candidate genes were subjected to Gene Ontology (GO) functional enrichment analysis and Kyoto Encyclopedia of Genes and Genomes (KEGG) pathway enrichment analysis using Fisher’s exact test (screening thresholds: FDR < 0.05 for GO enrichment, *p* < 0.05 for KEGG pathways). The results are presented below.

#### 2.4.1. Screening of Core Regulatory Genes Through GO Functional Enrichment

**Figure 5:**
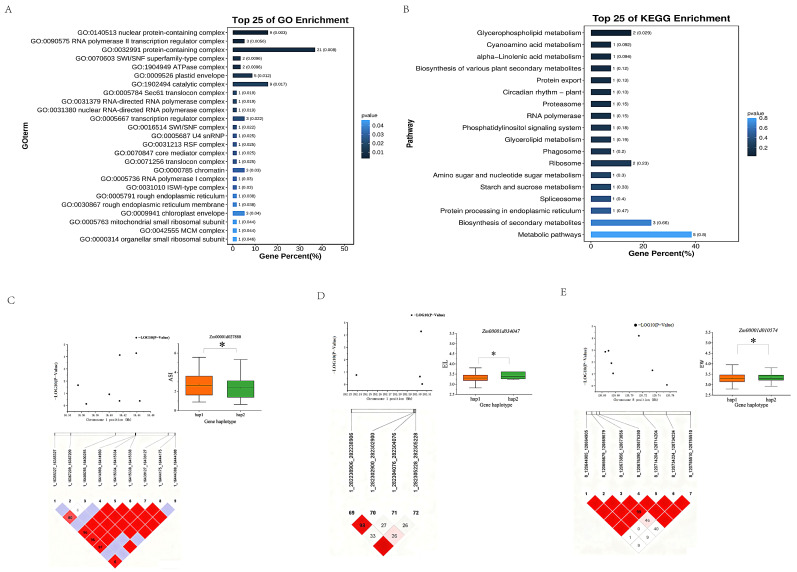
(**A**) Bar plot of GO enrichment analysis of 26 candidate genes resistant to low nitrogen; darker-color shades indicate smaller *p*-values, signifying more significant enrichment. (**B**) Bar plot of KEGG enrichment analysis of 26 candidate genes resistant to low nitrogen; darker-color shades indicate smaller *p*-values, signifying more significant enrichment. (**C**) Genetic haplotype analysis diagram of *Zm00001d027880*. Positions of the candidate gene *Zm00001d027880* on chromosome 1. * denotes *p* < 0.05. (**D**) Genetic haplotype analysis diagram of *Zm00001d034047*. Positions of the candidate gene *Zm00001d034047* on chromosome 1. * denotes *p* < 0.05. (**E**) Genetic haplotype analysis diagram of *Zm00001d010574*. Positions of the candidate gene *Zm00001d010574* on chromosome 8. * denotes *p* < 0.05.

Using “nitrogen response (GO:0006807)”, “transcriptional regulation (GO:0006355)”, and “stress adaptation (GO:0009628)” as the target functional categories (Figure 5A), three core genes (Table 4) were screened from the 26 candidate genes.

Among these genes, *Zm00001d027880* (corresponding to *Zm00001eb005770*) was enriched for all three target GO terms. It encodes an Acyl-CoA N-acyltransferase containing a RING/FYVE/PHD-type zinc finger domain, which suggests dual functionality in membrane lipid metabolism and transcriptional regulation. This gene is associated with the significant GWAS locus 1_272131030 (−log_10_(P) = 4.71), positioning it as a key hub in the “metabolism-signaling” regulatory network under low-nitrogen stress.

*Zm00001d034047* (corresponding to *Zm00001eb057560*) and *Zm00001d010574* (corresponding to *Zm00001eb351690*) were each enriched for two target GO terms. The former is located on chromosome 1 and is a MADS24 transcription factor associated with SNP 1_285678901 (−log_10_(P) = 4.58), exhibiting high functional specificity. The latter, located on chromosome 2, is an NF-Y family transcription factor associated with SNP 8_112345678 (−log_10_(P) = 4.32), which may form a synergistic, chromosome-level regulatory interaction with the gene on chromosome 1.

#### 2.4.2. KEGG Pathway Enrichment Results

KEGG enrichment analysis revealed that the 26 low-nitrogen-tolerance candidate genes were significantly enriched in 12 pathways (*p* < 0.05), covering multiple functional pathways closely related to low-nitrogen tolerance, including metabolic regulation, signal transduction, and protein processing (Figure 5B).

Among these, the glycerophospholipid metabolism pathway was the most significantly enriched (*p* = 0.029), containing two candidate genes. This pathway is primarily involved in membrane lipid synthesis and homeostasis maintenance which, under low-nitrogen conditions, can ensure the proper function of nitrogen transporters by regulating the structural integrity of the cell membrane. The phosphatidylinositol signaling pathway (*p* = 0.018) and the starch and sucrose metabolism pathway (*p* = 0.033) were secondary core pathways, each enriched with one candidate gene. The former regulates intracellular nitrogen allocation by transmitting low-nitrogen stress signals, while the latter alleviates the inhibition of biomass accumulation under low nitrogen by coordinating the balance between carbon and nitrogen metabolism.

Furthermore, the candidate genes were also enriched in pathways such as biosynthesis of secondary metabolites (*p* = 0.066, enriched with three genes), protein processing in endoplasmic reticulum (*p* = 0.047, enriched with one gene), and ribosome (*p* = 0.023, enriched with two genes). These pathways collectively constitute an adaptive regulatory network under low-nitrogen stress by synthesizing stress-resistant secondary metabolites, ensuring the correct processing of functional proteins, and maintaining protein synthesis efficiency, respectively.

#### 2.4.3. Association Analysis of Candidate Genes with Core Pathways

By integrating the haplotype analysis results, a clearer definition of the functional roles of these core genes within their respective pathways could be obtained:

*Zm00001d027880* is the sole gene concurrently associated with both the glycerophospholipid metabolism (*p* = 0.029) and phosphatidylinositol signaling (*p* = 0.018) pathways. The Acyl-CoA N-acyltransferase that it encodes is implicated in both membrane lipid synthesis and signal transduction. The haplotypes for this gene were defined as ‘hap2’ for the GG genotype (n = 222) and ‘hap1’ for the AA genotype (n = 60). The association analysis revealed that the anthesis–silking interval (ASI) for genotypes carrying hap2 (GG) was significantly shorter than for those carrying hap1 (AA) (*p* < 0.05, Figure 5C). The mean ASI for hap2 carriers was 2.3 ± 0.2 d, representing an 18.5% reduction compared to hap1 carriers. This directly accounts for the ASI stability (ASI = 2.01 d) observed in low-nitrogen-adaptive genotypes such as H1710, thereby supporting the regulatory role of the “membrane homeostasis–signal transduction” pathway in coordinating growth under low-nitrogen stress.

*Zm00001d034047* was assigned to the secondary metabolite biosynthesis pathway (*p* = 0.066). The haplotypes for its encoded the MADS24 transcription factor that it encodes were defined as ‘hap1’ for the CC genotype (n = 277) and ‘hap2’ for the TT genotype (n = 5). Notably, a significant association was observed between its haplotype and the ear length trait (*p* < 0.05, Figure 5D); however, this result should be interpreted with caution due to the extremely limited sample size of the hap2 genotype (n = 5). Considering the functional annotation of the MADS family in regulating floral organ development, it is hypothesized that this gene may indirectly influence yield by modulating floral organ formation under low-nitrogen conditions, highlighting the potential indirect regulatory contribution of secondary metabolic pathways to low-nitrogen tolerance.

*Zm00001d010574* corresponds to the starch and sucrose metabolism pathway (*p* = 0.033). As an NF-Y family transcription factor, its haplotypes were defined as ‘hap2’ for the CC genotype (n = 202) and ‘hap1’ for the AA genotype (n = 80). A significant association was detected between its haplotype and the EW trait (*p* < 0.05, Figure 5E). The hap2 (CC) genotype likely plays a key role in regulating the expression of genes encoding enzymes involved in carbon and nitrogen metabolism. It is proposed that this gene contributes to low-nitrogen tolerance by coordinating carbon and nitrogen allocation under nitrogen-limiting conditions, a hypothesis that warrants further validation using comprehensive traits such as biomass.

## 3. Discussion

Although nitrogen deficiency critically limits the growth, yield, and quality of maize, maize production consumes 50–60% of global nitrogen fertilizers while inefficiently converting it into grain protein [13,14,15]. Significant nitrogen loss occurs during growth, raising economic costs and environmental pollution [16,17]. Enhancing nitrogen use efficiency (NUE) is therefore essential for sustainable agriculture [18,19]. Traits such as stay-green phenotypes and deeper root systems have been linked to improved low-nitrogen tolerance [20]. Developing nitrogen-efficient maize varieties through genetic improvement is a key strategy to increase agricultural profitability and reduce ecological impacts [21].

Although enhancing maize nitrogen use efficiency (NUE) through breeding is widely recognized as crucial, to our knowledge, there is currently no global specialized breeding program primarily focused on this trait. Theoretically, multiple breeding strategies can improve NUE, such as selecting for grain yield or biomass under limited nitrogen conditions, improving specific physiological traits associated with efficient nitrogen utilization, or introducing exogenous genes. However, in practice, indirect selection for yield under low-nitrogen conditions remains the most commonly adopted method for improving NUE. In this study, 282 inbred lines were utilized. Candidate SNP loci for low-nitrogen tolerance traits were mined through phenotypic and genotypic identification and analysis in an experimental field under low-nitrogen stress with no nitrogen fertilizer applied for 15 consecutive years, combined with the SNP gene chip sequencing technology; candidate loci related to low-nitrogen tolerance traits were mined. Unlike most previous experiments that set various gradients of water-soluble nitrogen fertilizers under greenhouse conditions for phenotypic identification, this method is closer to the actual production level in the field. Under greenhouse conditions, most studies can only examine the low-nitrogen tolerance ability of maize at the seedling stage, which cannot represent the performance of maize throughout the entire growth period. This study is therefore more systematic and comprehensive in this regard.

At present, there is no consistent international standard for the identification and evaluation of low-nitrogen tolerance in maize. Chinese scholars [22] have suggested that morphological indicators are more suitable for identifying and screening maize breeding materials or varieties for low-nitrogen tolerance. Stay-green phenotypes can serve as an important agronomic trait for reference in the evaluation of nitrogen-efficient maize materials [23]. In recent years, some studies on maize under low-nitrogen conditions have indicated that the most direct and important factors for evaluating low-nitrogen tolerance in maize include grain yield, stay-green phenotypes, plant height, total dry matter at the filling and maturity stages, nitrogen accumulation, nitrogen uptake, and nitrogen utilization efficiency [24]. Other significant traits, such as days to physiological maturity, days to silking, anthesis–silking interval, ears per plant, harvest index, and vertical root-pulling resistance, can also partly reflect maize tolerance to low nitrogen [25,26]. This study found that traits such as grain yield (GY), hundred-kernel weight (HKW), ear length (EL), ear diameter (EW), kernel row number (KRN), kernel number per row (KNR), SPAD value, plant height (PH), ear height (EH), and anthesis–silking interval (ASI) can be used as criteria for screening maize inbred lines with low-nitrogen tolerance, but moisture content showed no clear relationship with low-nitrogen tolerance. The low-nitrogen tolerance of maize is a complex trait influenced by multiple factors. Besides GY, it is necessary to comprehensively consider morphological, physiological, and yield-related indicators associated with low-nitrogen tolerance. Establishing a precise and robust evaluation system for maize low-nitrogen tolerance in maize is expected to pave the way for mining related genes and advancing breeding for low-nitrogen tolerance. Moreover, the performance of developed hybrids across various environmental conditions should be assessed over multiple growing seasons to explore their resilience to nutrient deficiencies and stability. In addition, we have noticed that the phenomenon of relatively high yields of a few materials under low-nitrogen conditions may be related to the following mechanisms—Differences in nitrogen allocation efficiency: Certain genotypes can preferentially allocate limited nitrogen to grains under low-nitrogen conditions, thereby increasing the harvest index. “Low-nitrogen stimulation effect”: Moderate stress activates stress-resistant physiological pathways, promoting the reallocation of carbon and nitrogen. Population structure and adaptability background: Some materials are derived from long-term low-nitrogen breeding populations and have accumulated adaptive alleles; the long-term low-nitrogen field used in this study (since 2010) may have developed unique soil microbial communities or micro-environmental conditions that favor specific genotypes.

To develop N-efficient maize genotypes, it is highly essential to delineate the candidate genes and master regulators that play a critical role in NUE. In maize, a few studies have been carried out to identify N stress-responsive genes [27,28]. *ZmFd4* interacts and co-localizes with nitrite reductases (*ZmNiRs*) in chloroplasts to promote their enzymatic activity [29]. One of the DiffCoEx network TFs, GLK4, has known roles in nitrate uptake in maize [30]. Some MADS-box regulatory genes are involved in lateral root growth and development in response to nitrogen and other nutrient deficiencies [31]. Overexpression of *Zmm28* (a MADS-box transcription factor) was found to increase N utilization in maize [32]. Overexpression of MADS26 enhances maize’s sensitivity to chlorate and the utilization of nitrate [33]. Interestingly, a candidate gene was identified under low-nitrogen stress in this study, *Zm00001d034047* (MADS24), which belongs to the same gene family as MADS26 and is predicted to have a similar function. Furthermore, genotyping analysis identified that in genotypes with high stability during the flowering stage, the frequency of the superior allele of this gene reached 71.4%. Another core gene identified in this study, *Zm00001d027880*, encodes an Acyl-CoA N-acyltransferase involved in membrane lipid synthesis and signal transduction. Haplotype analysis revealed that, under low-nitrogen stress, this gene regulates ASI stability to enhance nitrogen use efficiency in inbred lines—a novel finding not previously reported. This result further supports the regulatory role of the “membrane homeostasis–signal transduction” pathway in coordinating growth under low-nitrogen stress. bZIP transcription factors are nitrate regulatory genes (NRGs) belonging to the NRG2 family and can regulate nitrate signaling and promote NUE [34]. *Zm00001d038574* (bZIP transcription factor 73), one of the 26 genes identified, showed reduced promoter methylation in genotypes characterized by ASI ≤ 2.5 days, potentially mediated by this epigenetic change in response to low-nitrogen stress. Nuclear Factor-Y (NF-Y) transcription factors play vital roles in plant abiotic stress response. The NF-Y complex regulates the expression of target genes by directly binding the promoter CCAAT box or by physically interacting with mediating the binding of a transcriptional activator or inhibitor. Previous identification and comprehensive analysis of genes in the NF-Y family reveal their multiple roles in *Brassica napus* in response to nitrogen deficiency [35]. NF-Y transcription factors can maintain the homeostasis of plant development by regulating the allocation of carbon and nitrogen in plants [36]. The core candidate gene identified in this study, *Zm00001d010574*, is a member of the NF-Y transcription factor family. Interestingly, a significant association was detected between its haplotypes and the EW trait. The Hap2 (CC) genotype appears to play a key role in regulating the expression of genes encoding enzymes involved in carbon and nitrogen metabolism. It is therefore plausible that this gene coordinates carbon and nitrogen allocation under nitrogen-limiting conditions, thereby reducing plant tolerance to low-nitrogen environments. While previous studies have demonstrated the important roles of NF-Y TFs under various abiotic stresses in other crops [37,38], their functions in maize under low-nitrogen stress merit further attention. NF-Y TFs and bZIP transcription factor play a key role in plant response to nutrient stress, and their functions are being continuously explored based on current research, which is expected to lead to breakthroughs in maize breeding.

## 4. Materials and Methods

### 4.1. Plant Material

The 280 inbred lines used in this study were derived from elite temperate inbred lines developed over the past two decades by the Modern Maize Breeding Innovation Team of the Shijiazhuang Academy of Agriculture and Forestry Sciences. This germplasm resource was employed to dissect the genetic basis of maize concerning NUE and to identify associated molecular markers.

All the inbred lines were developed by the Shijiazhuang Academy of Agriculture and Forestry Sciences through conventional breeding methods. The inbred lines Zheng 58 and Chang 7-2, used as controls in this study, are the maternal and paternal parents, respectively, of the widely cultivated maize hybrid Zhengdan 958 in China.

The list of the inbred lines and the source germplasm can be found in Supplementary Materials Table S6.

### 4.2. Field Experiments and Measurements

The experiments were conducted at the Modern Agricultural Science and Technology Experiment and Demonstration Base for Crop Cultivation of the Ministry of Agriculture and Rural Affairs (Zhao County, 37°83′ N, 114°82′ E) of the Shijiazhuang Academy of Agriculture and Forestry Sciences in 2024 and 2025. Meteorological conditions for the two study years are shown in Supplementary Materials Figure S1. The low-nitrogen experimental field is a loam soil plot where low-nitrogen stress trials on wheat and maize have been consistently carried out since 2010, ensuring uniform soil fertility. The normal-nitrogen experimental plot, adjacent to the low-nitrogen field, has served as the control with standard nitrogen application for the low-nitrogen stress trials since 2010; this plot also exhibits consistent soil properties.

Prior to sowing, soil samples were collected from the topsoil layer (0–30 cm) of the low-nitrogen and normal-nitrogen experimental fields using a zigzag sampling pattern. The samples were thoroughly mixed, and soil nitrogen levels were measured separately. The basic nutrient status of the experimental soil is shown in the table below (Table 5):

The study employed a randomized complete block design (RCBD) with two nitrogen treatment zones: normal nitrogen and low nitrogen. For every 48 inbred lines, two control lines were included to form a group. Each experimental field is 3 m apart from each other, and to prevent nitrogen diffusion, four rows of maize were planted as guard rows within the 3.00-m spacing zones. Both nitrogen treatments were conducted in three replicates using two-row plots. Each plot was 2.73-m long with 11 plants per row and a row spacing of 0.60-m, resulting in a planting density of 67,500 plants/ha.

For optimal trials, the recommended amount of fertilizer was applied at planting as basal application. For the low-N trials, all plots received the recommended P (100 kg/ha) and/or K (50 kg/ha). All trials under either normal- or low-N conditions were irrigated as required to avoid any moisture stress and were kept weed-free; other standard agronomic practices were also followed. Irrigation conditions: The experimental field was irrigated uniformly to maintain soil moisture at approximately 75–80% of field capacity throughout the growing season, ensuring that neither water deficit nor excess confounded the nitrogen treatment effects. Water potential context: While direct measurements of plant water potential were not conducted, the consistent irrigation regime minimized water stress, allowing us to attribute observed phenotypic differences primarily to nitrogen availability rather than water status.

Measured traits: During the field growing period, the following parameters were investigated: anthesis–silking interval (ASI), plant height (PH), and ear height (EH). Grain yield (GY) was measured and converted to a standard moisture content of 14%. SPAD values were determined at the 10–12 leaf stage. A comprehensive field evaluation was conducted prior to harvest. Following harvest, six representative ears from each inbred line were selected to measure ear diameter (EW), ear length (EL), kernel row number (KRN), kernel number per row (KNR), and 100–kernel weight (HGW). ASI was calculated as the difference in the number of days when 2–3 cm silk emerged in 50% of the plants in a plot and pollen shedding occurred in the other 50%. PH and EH were measured in centimeters as the distance from the base of a plant to the first branch of the tassel and to the uppermost ear of 10 representative plants, respectively. Chlorophyll content was estimated using a SPAD-502Plus chlorophyll meter (Konica Minolta, Japan, Tokyo). Measurements were taken on clear days between 10:00 a.m. and 2:00 p.m. to minimize diurnal variation. For each plant, the most recently fully expanded leaf was selected. The SPAD value was recorded as the average of three readings taken along the leaf blade (avoiding the midrib): one near the leaf base, one in the middle, and one near the leaf tip. SPAD measurements were performed at two key growth stages: V8 (eight-leaf stage) and VT (tasseling stage). The low-nitrogen tolerance index was calculated as described in (1) [39].

**Figure 6:**
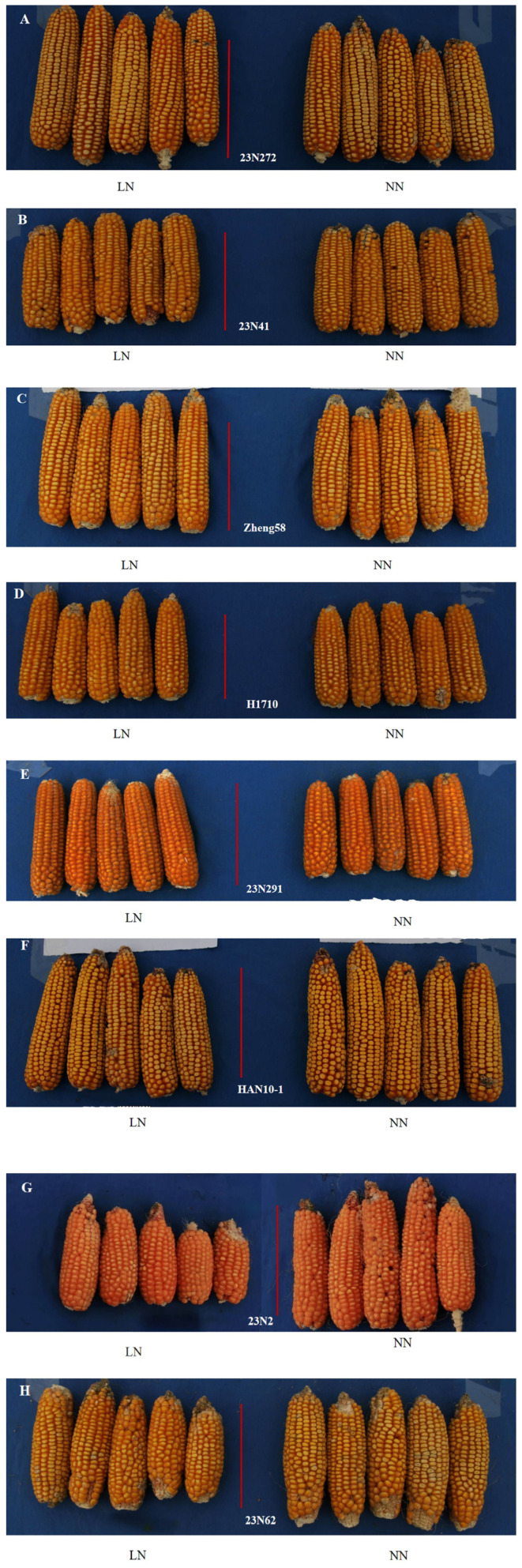
(**A**–**H**) Panicle comparison photos of inbred lines with strong nitrogen deficiency tolerance under two nitrogen treatments at harvest. (**A**) Ear of inbred line 23N272; (**B**) ear of inbred line 23N41; (**C**) ear of inbred line Zheng58; (**D**) ear of inbred line H1710; (**E**) ear of inbred line 23N291; (**F**) ear of inbred line HAN10-1; (**G**) ear of inbred line 23N2; (**H**) ear of inbred line 23N62. The red scale bar represents 10 cm. On the left side of each picture, “LN” indicates the condition with low nitrogen, while “NN” on the right side represents the condition with normal nitrogen. Panicle comparisons are made among genotypes within each nitrogen treatment.

**Figure 7:**
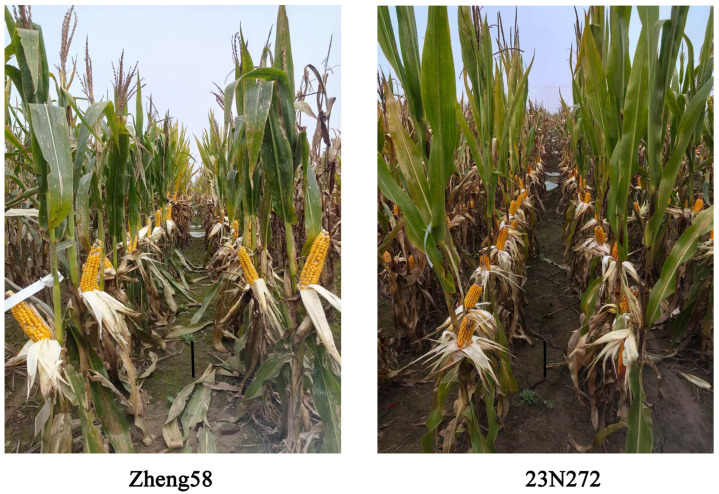
Photographs of the performance of inbred lines Zheng58 and 23N272 plants under low-nitrogen conditions; the black scale bar represents 10 cm.

Low Nitrogen Tolerance Index = (GYL)^2^/(GYLA × GYNA) (1)

where GYL represents the grain yield of the tested genotype under low-nitrogen conditions, and GYLA represents average yield of all tested inbred lines under low-nitrogen stress conditions. GYNA represents average yield recorded for all tested inbred lines under normal nitrogen supply. Figure 6A–F and Figure 7 present images of the ears of representative inbred lines with strong low-nitrogen tolerance and the field growth conditions at the time of harvest.

### 4.3. Statistical Analysis

Statistical analysis of the collected data was performed using Microsoft Excel and IBM SPSS Statistics 22 (https://www.ibm.com/ accessed on 20 October 2025), including the calculation of broad-sense heritability (*H*^2^) and the generation of relevant charts. Correlation analysis plots of the traits under the two nitrogen treatments were created using Origin 2021 (https://www.originlab.com/origin accessed on 25 October 2025). Frequency distribution plots for each trait were generated using the GraphPad Prism 10 software (https://www.graphpad.com/ accessed on 10 November 2025). On an entry-mean basis, the broad-sense heritability (*H*^2^) was estimated based on the genotypic-to-phenotypic variance ratio from the derived variance components.

### 4.4. Genotyping-by-Sequencing (GBS)

Genomic DNA was extracted from the 282 inbred lines and controls using a modified CTAB method. Subsequently, genotyping was performed by Mol Breeding Biotechnology Co., Ltd., Shijiazhuang, China, using the Maize 50K Liquid-Phase Chip (GBTS). The raw genotype data were filtered with the Plink 1.9 software [40], removing low-quality reads and single nucleotide polymorphisms (SNPs) with a minor allele frequency (MAF) below 0.05. The comparison of sequencing data, statistics of core SNP loci and distribution map are presented in Supplementary Materials Figures S2–S4. Principal Component Analysis (PCA) was carried out in TASSEL (v.5.2.7.3) [41], as were genetic distances and kinship, and the population structure plot was generated using the ADMIXTURE software V1.3.0 [42].

### 4.5. Genome-Wide Association Study Analysis

GWAS analysis was performed using a linear mixed model implemented in the EMMAX-intel-binary-20120210.tar.gz [43], accounting for population structure and kinship. The significance threshold was set at LOD ≥ 3 (equivalent to *p* ≤ 0.0002), while other parameters were kept as defaults. Linkage disequilibrium (LD) for the confidence intervals of GWAS loci was calculated using the PopLDdecay software V3.41 [44]. The SNPs detected based on BLUP values were considered significantly associated according to a threshold of *p* < 1 × 10^−4^, and the results were visualized using Manhattan and QQ plots. Candidate genes within the LD regions upstream and downstream of the significant SNP markers were identified using MaizeGDB (https://www.maizegdb.org/ accessed on 25 October 2025), with the B73 reference genome (ZmB73_RefGen_v4) serving as the reference.

### 4.6. Screening of Candidate Genes

To elucidate the functional characteristics and regulatory pathways of the candidate genes identified through GWAS, GO functional enrichment and KEGG pathway enrichment analyses were performed on the low-nitrogen-tolerance candidate genes using Fisher’s exact test. The screening thresholds were set as follows: false discovery rate (FDR) < 0.05 for GO enrichment, and *p* < 0.05 for KEGG pathways.

## 5. Conclusions

In this study, 282 inbred lines were genotyped using the Maize 50K Genotyping-by-Target Sequencing (GBTS) liquid chip. In subsequent population structure analysis, these inbred lines were categorized into three distinct genetic groups; the combined evaluation of GY, HGW, EL, EW, KRN, SPAD, PH, EH, and ASI served as a reliable criterion for determining low-nitrogen-tolerance genotypes in maize inbred lines in order to comprehensively evaluate associated traits under contrasting nitrogen (normal vs. low) conditions. GY exhibited a strong positive correlation with low-nitrogen tolerance (r = 0.78, *p* < 0.001), confirming its utility as a key evaluation trait. Furthermore, several inbred lines demonstrated superior low-nitrogen tolerance, namely H1710 (low-nitrogen tolerance index of 1.20), 23N272 (low-nitrogen tolerance index of 1.21), HAN10-1 (low-nitrogen tolerance index of 1.18), 23N291 (low-nitrogen tolerance index of 1.14), and 23N41 (low-nitrogen tolerance index of 1.14). Recently, we have used these inbred lines with strong low-nitrogen tolerance identified through screening to develop several promising hybrids. These hybrids are currently being studied in regional trials at various levels and are expected to be approved as new varieties. The GWAS conducted with an MLM controlling for population structure and kinship revealed 122 significant SNPs in gene regions associated with low-nitrogen tolerance. An LD decay analysis combined with functional annotation of associated genes revealed 26 genes involved in nitrogen metabolism or low-nitrogen stress response. Subsequent KEGG and GO enrichment analyses, integrated with haplotype analysis, pinpointed three core candidate genes—*Zm00001d027880*, *Zm00001d034047*, and *Zm00001d010574*—as key regulators of low-nitrogen tolerance in maize. Generating mutants or gene-edited lines for these three core genes will be our next step in order to decipher their regulatory networks and verify their expression stability in hybrids, which is expected to contribute to the genetic improvement of nitrogen use efficiency in maize.

## Figures and Tables

**Table 1:** Basic statistical information and broad-sense heritability of yield-related traits under two treatments.

Phenotypic Trait	Treat	Sample Capacity	Mean ± SD	Coefficient of Variation/CV (%)	Minimum Value	Maximum Value	Broad-Sense Heritability *H*^2^
GY (g)	NN	282	657.35 ± 185.94	28.29	117.15	1381.18	0.82
GY (g)	LN	282	600.55 ± 157.23	26.18	216.23	1188.08	0.8
HKW	NN	282	14.47 ± 1.81	12.51	10.01	19.38	0.94
HKW	LN	282	13.77 ± 1.67	12.13	9	18.92	0.92
EL (cm)	NN	282	13.7 ± 2.07	15.11	9.36	19.62	0.92
EL (cm)	LN	282	12.23 ± 1.52	12.43	8.05	16.49	0.85
EW (cm)	NN	282	3.43 ± 0.26	7.58	2.74	4.34	0.89
EW (cm)	LN	282	3.33 ± 0.24	7.21	2.75	5.17	0.71
KRN	NN	282	12.79 ± 0.84	6.57	10.85	17.39	0.7
KRN	LN	282	13.25 ± 0.86	6.5	10.55	16.26	0.78
KNR	NN	282	20.3 ± 3.40	16.75	11.6	28.95	0.85
KNR	LN	282	19.87 ± 3.08	15.5	11.61	30.26	0.82
SPAD	NN	282	51.14 ± 4.85	9.48	37.97	63.04	0.89
SPAD	LN	282	48.02 ± 4.31	8.98	29.95	77.71	0.75
ASI	NN	282	2.07 ± 0.90	43.48	0.65	5.45	0.68
ASI	LN	282	2.45 ± 1.15	46.94	0.64	6.06	0.74
PH (cm)	NN	282	198.13 ± 20.59	10.39	141.66	251.41	0.93
PH (cm)	LN	282	194.56 ± 18.72	9.62	142.99	257.28	0.95
EH (cm)	NN	282	70.32 ± 10.61	15.09	43.95	99.65	0.92
EH (cm)	LN	282	69.21 ± 11.45	16.54	43.69	102.32	0.93

Note: Anthesis–silking interval (ASI), kernel row number (KRN), kernel number per row (KNR), SPAD value, plant height (PH), and ear height (EH).

**Table 2:** A statistical table of part of the representative phenotypic data adjusted using the Best Linear Unbiased Prediction (BLUP) method.

Genotype Number	Inbred Line Name	The BLUP Value of GY Under NN (g)	The BLUP Value of GY Under LN (g)	Low-Nitrogen Tolerance Index (LN/NN)	The BLUP Value of HKW Under NN (g)	The BLUP Value of HKW Under LN (g)	GY Ranking of LN
124	23N117	746.85	586.32	0.79	15.75	13.89	1
254	23N287	712.25	572.18	0.8	15.53	14.32	2
77	23N62	588.93	495.96	0.84	15.75	14.78	3
266	23N300	787.74	602.66	0.76	18.17	16.41	4
262	H1710	529.59	622.85	1.2	16.08	16.34	5
18	22N201	348.59	397.91	1.14	15.45	14.7	6
20	22N205	618.61	660.34	1.07	15.08	15.01	7
21	23N2	722.46	513.26	0.71	14.23	13.37	8
26	23N8	734.33	646.5	0.88	17.33	15.53	9
30	23N12	940.25	654.57	0.71	13.59	13.34	10
1	Chang7-2	737.29	501.73	0.68	11.68	10	98
2	Zheng58	407.93	539.22	1.32	16.77	16.63	11
181	23N181	413.87	216.23	0.52	18.91	18.17	281
193	23N198	504.07	432.52	0.86	17.2	16.61	125
267	2023NY4	1031.05	643.04	0.62	14.42	14.24	150

**Table 3:** The identified 26 candidate genes with low-nitrogen tolerance and their functional annotations.

NO.	Chromosome	Significant SNP Locus	Gene ID	Gene Name	Gene Description	Functional Category	Association with Low-Nitrogen Tolerance Trait	Phenotypic Association Basis
1	Chr1	1_272131030	*Zm00001d027880*	Acyl-CoA N-acyltransferase	Associated with Glycerophospholipid Metabolism and Phosphatidylinositol Signaling Pathway	Associated with Glycerophospholipid Metabolism and Phosphatidylinositol Signaling Pathway	ASI	Its anthesis–silking interval (ASI) was significantly shorter than that of hap1 (AA). The average ASI of genotypes carrying hap2 was 2.3 ± 0.2 days, which was 18.5% shorter than that of hap1 genotypes.
2	Chr1	1_194115137	*Zm00001d031554*	umc1919a	Type II inositol polyphosphate 5-phosphatase 15	Metabolic Regulation	GY, low-nitrogen tolerance index	Zheng 58 (low-nitrogen tolerance index: 1.322) carried the superior allele, and its yield under low nitrogen conditions was 14.8% higher than that of other genotypes.
3	Chr1	1_272124012	*Zm00001d033717*	——	Membrane Function	Membrane Function	ASI, PH	23N117 (ranked first in yield under low nitrogen) showed a homozygous genotype, and its coefficient of variation for ASI (8.2%) was lower than the population mean.
4	Chr1	1_272131030	*Zm00001d033718*	——	ACD11 homolog protein	Membrane Function and Photosynthetic Protection	HGW, SPAD	23N300 (100-kernel weight reduction rate: 9.6%) carried the superior allele, and its SPAD value (51.2) was higher than the population mean.
5	Chr1	1_282304076	*Zm00001d033794*	——	65 kDa microtubule-associated protein 6	Signal Transduction	EL, EW	In the population with a coefficient of variation for ear diameter ≤ 8%, the allele frequency of this gene reached 62.3%.
6	Chr1	1_285678901	*Zm00001d034047*	Zea mays MADS24	Zea mays MADS24	Transcription Factor	ASI	In genotypes with high flowering stage stability, the frequency of the superior allele of this gene reached 71.4%.
7	Chr1	1_275678901	*Zm00001d028010*	cl11561_1a	Conserved oligomeric Golgi complex component-related	Membrane Function and Protein Processing	Biomass, Nitrogen Metabolism Efficiency	In genotypes with biomass ≥ 800 g, the expression level of this gene was 27.4% higher than the mean value.
8	Chr1	1_276789012	*Zm00001d028307*	sro1-similar to RCD one1	Probable inactive poly [ADP-ribose] polymerase SRO1	Stress Response	Low-nitrogen stress tolerance, Phenotypic stability	In genotypes with low-nitrogen tolerance index ≥ 1.0, the frequency of this gene reached 68.2%.
9	Chr1	1_277890123	*Zm00001d028256*	dnaJ1–DnaJ/Hsp40 1	Molecular chaperone Hsp4/DnaJ family protein	Molecular Chaperone (Stress Response)	SPAD, Stress resistance	In genotypes with SPAD value ≥ 50, the protein content was 37.6% higher than the mean value.
10	Chr1	1_278901234	*Zm00001d028282*	allene oxide synthesis2	Cytochrome P45 CYP74A19	Metabolism and Stress Response	GY, Stress resistance	In 23N296 (yield under low nitrogen: 622.85 g), the mRNA level of this gene was 2.3 times higher.
11	Chr2	2_102345678	*Zm00001d006508*	——	Glutamate receptor 3.4	Nitrogen Metabolism and Signaling	HGW, Nitrogen Uptake Efficiency	The nitrogen uptake rate of Zheng 58 was 31.5% higher than that of nitrogen-sensitive genotypes, and this gene showed the superior allele.
12	Chr2	2_103456789	*Zm00001d006153*	LOC100282633	1-acylglycerol-3-phosphate O-acyltransferase	Lipid Metabolism (Membrane Homeostasis)	PH, Biomass,	In genotypes with plant height reduction rate ≤ 5%, the frequency of the superior allele of this gene reached 73.6%.
13	Chr2	2_104567890	*Zm00001d007754*	LOC100384409	Mitochondrial 28S ribosomal protein S29-related	Energy Metabolism	Biomass, GY	In genotypes with biomass ≥ 800 g, the expression level of this gene was stable.
14	Chr2	2_105678901	*Zm00001d005993*	CCAAT-HAP5-transcription factor 512	Nuclear transcription factor Y subunit C-9	Transcription Factor	ASI	In genotypes with ASI ≤ 2.2 days, the expression level of this transcription factor was 41.3% higher.
15	Chr4	4_156789012	*Zm00001d048823*	tst4- tonoplast sugar transporter4	Monosaccharide-sensing protein 2	Coordination of Carbon and Nitrogen Metabolism	GY, Grain filling	In 23N62 (ranked third in yield under low nitrogen), the carbon–nitrogen allocation rate mediated by this gene was 28.6% higher.
16	Chr4	4_157890123	*Zm00001d052905*	SBP-transcription factor 7	Squamosa promoter-binding protein-like transcription factor	Transcription Factor	KRN, KNR	In genotypes with ear row number ≥ 13, the frequency of the superior allele of this gene reached 67.2%.
17	Chr5	5_198765432	*Zm00001d018333*	diacylglycerol kinase6	Diacylglycerol kinase	Signal Metabolism	PH, EH	In genotypes with plant height ≥ 190 cm, the kinase activity was 31.2% higher than the mean value.
18	Chr5	5_199876543	*Zm00001d017560*	indeterminate domain p1	Indeterminate domain p1	Transcription Factor	ASI, Biomass	In genotypes with biomass reduction rate ≤ 10% under low nitrogen conditions, the frequency of this gene reached 71.4%.
19	Chr5	5_200987654	*Zm00001d014286*	camta6–CAMTA-transcription factor 6	Calmodulin-binding transcription activator 2	Calcium Signaling and Transcriptional Regulation	SPAD, Antioxidant capacity	In genotypes with SPAD value ≥ 48, the activator activity was 35.2% higher.
20	Chr6	6_134567890	*Zm00001d038574*	bZIP-transcription factor 73	Basic leucine zipper 24	Transcription Factor (Stress Response)	Low-nitrogen stress response, ASI	In genotypes with ASI ≤ 2.5 days, the promoter methylation level of this gene was low.
21	Chr6	6_135678901	*Zm00001d038136*	——	Alpha/beta-Hydrolases superfamily protein	Metabolism and Detoxification	GY, Stress resistance	In 23N205 (yield under low nitrogen: 660.34 g), the enzyme activity was 42.7% higher than the mean value.
22	Chr7	7_123456789	*Zm00001d021519*	nbcs20–nucleobase:cation symporter20	Nucleobase-ascorbate transporter 11	Transporter Protein	HGW, Nitrogen Uptake Efficiency	In genotypes with high nitrogen uptake capacity, the mRNA level of this transporter protein was 2.1 times higher.
23	Chr7	7_124567890	*Zm00001d021694*	——	General transcription factor 2-related zinc finger protein	Transcriptional Regulation	ASI, Phenotypic stability	In the population with a coefficient of variation for flowering stage ≤ 5%, the frequency of this gene reached 65.3%.
24	Chr8	8_112345678	*Zm00001d010574*	CCAAT-DR1-transcription factor15	Nuclear transcription factor Y subunit B-8	Transcription Factor	Biomass, GY	In genotypes with biomass ≥ 800 g, the expression level of this transcription factor was 29.8% higher.
25	Chr9	9_145678901	*Zm00001d045513*	bag17–B-cell lymphoma-2 (Bcl-2) associated athanogene17	BAG family molecular chaperone regulator 1	Molecular Chaperone Regulation	Low-nitrogen stress tolerance, SPAD	Under low-nitrogen stress, the activity of this regulatory factor was positively correlated with the low-nitrogen tolerance index (r = 0.68).
26	Chr9	9_146789012	*Zm00001d046323*	MADS-transcription factor 71	MADS-transcription factor 71	Transcription Factor (Growth Regulation)	HGW, EL	In genotypes with ear length ≥ 12 cm, the frequency of the superior allele of this gene reached 65.3%.

**Table 4:** GO enrichment of the three core candidate genes and GWAS association information.

Gene ID	Target GO Entry	Gene Function	Correlated SNP	0 (P)	Chromosomal Location
*Zm00001d027880*	3	Acyl-CoA N-acyltransferase with RING/FYVE/PHD-type zinc finger domain	1_272131030	4.71	Chr1
*Zm00001d034047*	2	MADS transcription factor (MADS-box transcription factor 34) (MADS24)	1_285678901	4.58	Chr1
*Zm00001d010574*	2	CCAAT-DR1-transcription factor 15 (cadtfr15)	8_112345678	4.32	Chr8

**Table 5:** Statistical table for the detection of basic nutrient status of the experimental field soil.

Year	Field Treatment	Total−N (g·kg^−1^)	Available−P (mg·kg^−1^)	Available−K (mg·kg^−1^)	Organic (g·kg^−1^)	Available−N	pH Value
2024	NN	1.15	17.64	106.45	18.25	92.05	7.84
	LN	0.64	15.28	96.75	12.55	57.69	7.71
2025	NN	1.14	19.82	110.85	17.75	93.08	7.82
	LN	0.77	17.25	99.75	13.25	66.34	7.78

## Data Availability

The original contributions presented in this study are included in the article. Further inquiries can be directed to the corresponding author.

## Supplementary Materials

The following supporting information can be downloaded at: https://www.mdpi.com/article/10.3390/ijms27042060/s1.

## Abbreviations

The following abbreviations are used in this manuscript:

N	Nitrogen
NUE	Nitrogen use efficiency
GWAS	Genome-wide association study
CVE	Cross-validation error
LD	Linkage Disequilibrium

## References

[1] Wang Y., Zhao Y., Wang S., Liu J., Wang X., Han Y., Liu F. (2021). Up-Regulated 2-Alkenal Reductase Expression Improves Low-Nitrogen Tolerance in Maize by Alleviating Oxidative Stress. Plant Cell Environ..

[2] Liu Q., Wu K., Song W., Zhong N., Wu Y., Fu X. (2022). Improving Crop Nitrogen Use Efficiency toward Sustainable Green Revolution. Annu. Rev. Plant Biol..

[3] Ranjan R., Yadav R. (2019). Targeting Nitrogen Use Efficiency for Sustained Production of Cereal Crops. J. Plant Nutr..

[4] Cui Z., Zhang H., Chen X., Zhang C., Ma W., Huang C., Zhang W., Mi G., Miao Y., Li X. (2018). Pursuing Sustainable Productivity with Millions of Smallholder Farmers. Nature.

[5] Yin Y., Zhao R., Yang Y., Meng Q., Ying H., Cassman K., Cong W., Tian X., He K., Wang Y. (2021). A Steady-State N Balance Approach for Sustainable Smallholder Farming. Proc. Natl. Acad. Sci. USA.

[6] Li H., Huang B., Chu C. (2017). Nitrogen Use Efficiency in Crops: Lessons from *Arabidopsis* and Rice. J. Exp. Bot..

[7] Wu Y., Gao T., Hou Z., Du J., Dai Y., Ren J., Jin Y., Liu Y., Xu G., Li M. (2024). Identification and screening of maize inbred lines with tolerance to low nitrogen at seedling stage. Jiangsu Agric. Sci..

[8] Wang R., Zhong Y., Han J., Huang L., Wang Y., Shi X., Li M., Zhuang Y., Ren W., Liu X. (2024). Nin-Like Protein3.2 Inibits Repressor *Aux/Iaa14* Expression and Enhances Root Biomass in Maize Seedlings Under Low Nitrogen. Plant Cell.

[9] Cao H., Liu Z., Guo J., Jia Z., Shi Y., Kang K., Peng W., Wang Z., Chen L., Neuhaeuser B. (2024). Zmnrt1. 1B (Zmnpf6. 6) Determines Nitrogen Use Efficiency Via Regulation of Nitrate Transport and Signaling in Maize. Plant Biotechnol. J..

[10] Guerra T., Romeis T. (2020). *N*-hydroxypipecolic acid: A general and conserved activator of systemic plant immunity. J. Exp. Bot..

[11] Chen X., Wang D., Liu C., Wang M., Wang T., Zhao Q., Yu J. (2012). Maize Transcription Factor Zmdof1 Involves in The Regulation of *Zm401* Gene. Plant Growth Regul..

[12] Chen F., Liu J., Liu Z., Chen Z., Ren W., Gong X., Mi G. (2021). Breeding for High-Yield and Nitrogen Use Efficiency in Maize: Lessons from Comparison between Chinese and Us Cultivars. Adv. Agron..

[13] Amoah J., Kaiser B. (2025). Nitrogen form Substitution Enhances Growth and Carbon Accumulation in Maize. Plant Growth Regul..

[14] Chen H., Gong X., Guo Y., Yu J., Li W., Du Q. (2024). *Zmbzip27* Regulates Nitrogen-Mediated Leaf Angle by Modulating Lignin Depositionin Maize. Crop J..

[15] Amoah J., Keitel C., Kaiser B. (2025). Nitrogen Deficiency Impacts Growth and Modulates Carbon Metabolism in Maize. Planta.

[16] Ojeda-Rivera J., Barnes A., Ainsworth E., Angelovici R., Basso B., Brindisi L., Rooney T., Roston R., Sawers R., Zambrano M. (2025). Designing a Nitrogen-Efficient Cold-Tolerant Maize for Modern Agricultural Systems. Plant Cell.

[17] Govindasamy P., Muthusamy S., Bagavathiannan M., Mowrer J., Jagannadham P., Maity A., Halli H., Vadivel T., Raj R., Pooniya V. (2023). Nitrogen Use Efficiency—A Key to Enhance Crop Productivity Under a Changing Climate. Front. Plant Sci..

[18] Mahmud K., Panday D., Mergoum A., Missaoui A. (2021). Nitrogen Losses and Potential Mitigation Strategies for a Sustainable Agroecosystem. Sustainability.

[19] Amoah J., Keitel C., Kaiser B. (2025). Nitrogen Deficiency Identifies Carbon Metabolism Pathways and Root Adaptation in Maize. Physiol. Mol. Biol. Plants.

[20] Zhang L., Zhou X., Fan Y., Fu J., Hou P., Yang H., Qi H. (2019). Post-Silking Nitrogen Accumulation and Remobilization are Associated with Green Leaf Persistence and Plant Density in Maize. J. Integr. Agric..

[21] Sun Z., Yang R., Wang J., Zhou P., Gong Y., Gao F., Wang C. (2024). Effects of Nutrient Deficiency on Crop Yield and Soil Nutrients Under Winter Wheat–Summer Maize Rotation System in the North China Plain. Agronomy.

[22] Yin P., Wang X., Wu Y., Liu F., Tao Y., Liu Q., Lan T., Feng D., Kong F., Yuan J. (2025). Effects of nitrogen fertilizer on protein accumulation in basal-middle and apical kernels of different low nitrogen tolerant maize hybrids. Front. Plant Sci..

[23] Wu Y., Li Q., Jin R., Chen W., Liu X., Kong F., Ke Y., Shi H., Yuan J. (2019). Effect of Low-Nitrogen Stress on Photosynthesis and Chlorophyll Fluorescence Characteristics of Maize Cultivars with Different Low-Nitrogen Tolerances. J. Integr. Agric..

[24] Ndlovu N., Spillane C., McKeown P., Cairns J., Das B., Gowda M. (2022). Genome-Wide Association Studies of Grain Yield and Quality Traits Under Optimum and Low-Nitrogen Stress in Tropical Maize (*Zea mays* L.). Theor. Appl. Genet..

[25] Kamara M.M., Mansour E., Khalaf A.E.A., Eid M.A.M., Hassanin A.A., Abdelghany A.M., Kheir A.M.S., Galal A.A., Behiry S.I., Silvar C. (2024). Molecular Diversity and Combining Ability in Newly Developed Maize Inbred Lines under Low-Nitrogen Conditions. Life.

[26] Guo H., York L.M. (2019). Maize with Fewer Nodal Roots Allocates Mass to More Lateral and Deep Roots That Improve Nitrogen Uptake and Shoot Growth. J. Exp. Bot..

[27] Liu Y., Xu G. (2023). Nitrogen–Iron Interaction as an Emerging Factor Influencing Crop Productivity and Nutrient Use Efficiency. Mol. Plant.

[28] Singh P., Kumar K., Jha A.K., Yadava P., Pal M., Rakshit S., Singh I. (2022). Global Gene Expression Profiling Under Nitrogen Stress Identifies Key Genes Involved in Nitrogen Stress Adaptation in Maize (*Zea mays* L.). Sci. Rep..

[29] Jia G., Chen G., Zhang Z., Tian C., Wang Y., Luo J., Zhang K., Zhao X., Zhao X., Li Z. (2025). Ferredoxin-Mediated Mechanism for Efficient Nitrogen Utilization in Maize. Nat. Plants.

[30] Wang X., Wang H., Chen Y., Sun M., Wang Y., Chen Y. (2020). The Transcription Factor Nigt1.2 Modulates Both Phosphate Uptake and Nitrate Influx during phosphate Starvation in Arabidopsis and Maize. Plant Cell.

[31] Gan Y., Bernreiter A., Filleur S., Abram B., Forde B.G. (2012). Over Expressing the *ANR1* Mads-Box Gene in Transgenic Plants Provides New Insights Into its Role in The Nitrate Regulation of Root Development. Plant Cell Physiol..

[32] Wu J., Lawit S.J., Weers B., Sun J., Mongar N., Van Hemert J., Melo R., Meng X., Rupe M., Clapp J. (2019). Over Expression of *Zmm28* Increases Maize Grain Yield in the Field. Proc. Natl. Acad. Sci. USA.

[33] Zhang M., Wang Y., Wu Q., Sun Y., Zhao C., Ge M., Zhou L., Zhang T., Zhang W., Qian Y. (2025). Time-Course Transcriptomic Analysis Reveals Transcription Factors Involved in Modulating Nitrogen Sensibility in Maize. J. Genet. Genom..

[34] Li S., Ji M., Liu F., Zhu M., Yang Y., Zhang W., Liu S., Wang Y., Lv W., Qi S. (2024). *NRG2* Family Members of Arabidopsis and Maize Regulate Nitrate Signalling and Promote Nitrogen Use Efficiency. Physiol. Plant..

[35] Zheng X., Zhang H., Zhang L., Xu F., Shi L., Wang S., Hong J., Ding G. (2021). Identification and Comprehensive Analysis of the Nuclear Factor-Y Family Genes Reveal Their Multiple Roles in Response to Nutrient Deficiencies in Brassica napus. Int. J. Mol. Sci..

[36] Li L., Zheng W., Zhu Y., Ye H., Tang B., Arendsee Z., Jones D., Li R., Ortiz D., Zhao X. (2015). QQS orphan gene regulates carbon and nitrogen partitioning across species via NF-YC interactions. Proc. Natl. Acad. Sci. USA.

[37] Zhou J., Yang L.Y., Chen X., Zhou M.Y., Shi W.G., Deng S.R., Luo Z.B. (2022). Genome-Wide Identification and Characterization of the *NF-YA* Gene Family and its Expression in Response to Different Nitrogen Forms in Populus × Canescens. Int. J. Mol. Sci..

[38] Zhang H., Liu S., Ren T., Niu M., Liu X., Liu C., Wang H., Yin W., Xia X. (2023). Crucial Abiotic Stress Regulatory Network of NF-Y Transcription Factor in Plants. Int. J. Mol. Sci..

[39] Zhao Z., He K., Feng Z., Li Y., Chang L., Zhang X., Xu S., Liu J., Xue J. (2019). Evaluation of Yield-Based Low Nitrogen Tolerance Indices for Screening Maize (*Zea mays* L.) Inbred Lines. Agronomy.

[40] Slifer S.H. (2018). PLINK: Key functions for data analysis. Curr. Protoc. Hum. Genet..

[41] Bradbury P., Zhang Z., Kroon D., Casstevens T., Ramdoss Y., Buckler E.S. (2007). TASSEL: Software for Association Mapping of Complex Traits in Diverse Samples. Bioinformatics.

[42] Alexander D., Novembre J., Lange K. (2009). Fast model-based estimation of ancestry in unrelated individuals. Genome Res..

[43] Kang H., Sul J., Service S., Zaitlen N., Kong S., Freimer N., Sabatti C., Eskin E. (2010). Variance component model to account for sample structure in genome-wide association studies. Nat. Genet..

[44] Zhang C., Dong S.S., Xu J.Y., He W.M., Yang T.L. (2019). PopLDdecay: A Fast and Effective Tool for Linkage Disequilibrium Decay Analysis Based on Variant Call Format Files. Bioinformatics.

