# Supplementary material for: GWAS-Based Mining of Candidate Genes for Low-Nitrogen Tolerance in Maize
Source: Int J Mol Sci. 2026 Feb 23;27(4):2060. doi: 10.3390/ijms27042060 (PMC12940573; doi:10.3390/ijms27042060)
Supplement: Supplementary file 1 [file ijms-27-02060-s001.zip › ijms-4097181-supplementary.pdf]

Table S1. (ANOVA) result for phenotypic data in 2024 and 2025

Phenotype	Year effect（F value，p value）	Trait effect（F value，p value）	The interaction between the year and the treatment（F value，p value）	Significance summary
GY	0.231, p=0.631	121.35, p<0.001***	0.029, p=0.865	Handle extremely significant matters（LN decline）
HGW	2.13, p=0.145	38.45, p<0.001***	0.85, p=0.357	Handle extremely significant matters（LN decline）
EL	0.002, p=0.962	76.21, p<0.001***	0.07, p=0.789	Handle extremely significant matters（LN Shorten the ear length）
EW	0.014, p=0.905	9.87, p=0.002**	0.18, p=0.673	significantly（LN Reduce the EW）
KRN	0.241, p=0.624	24.56, p<0.001***	0.003, p=0.957	Handle extremely significant matters（LN Reduce the KRN）
KNR	0.105, p=0.746	67.34, p<0.001***	0.011, p=0.918	Handle extremely significant matters（LN Reduce the KNR）
SPAD	0.012, p=0.914	89.12, p<0.001***	0.034, p=0.854	Handle extremely significant matters（LN Reduce the SPAD）
ASI	0.085, p=0.771	45.67, p<0.001***	0.012, p=0.913	Handle extremely significant matters（LN Extend the ASI）
PH	0.321, p=0.571	18.23, p<0.001***	0.221, p=0.638	Handle extremely significant matters（LN Reduce the PH）
EH	0.154, p=0.695	36.45, p<0.001***	0.067, p=0.796	Handle extremely significant matters（LN Reduce the EH）

Note: *** show p<0.001，** show p<0.01，* show p<0.05

Table S2. Quality statistics of sequencing data

Sample	Raw Reads Number	Clean Reads Number	Raw Bases(bp)	Clean Bases(bp)	Effective Rate(%)	Q20 (%)	Q30 (%)
1	11285004	11285004	1692750600	1640333598	96.9	98.97	96.55
2	10769062	10769062	1615359300	1573704572	97.42	98.92	96.39
3	10275128	10275128	1541269200	1501697770	97.43	98.83	96.08
4	12700658	12700658	1905098700	1850435646	97.13	98.96	96.56
5	12235496	12235496	1835324400	1783223226	97.16	98.99	96.61
6	10061862	10061862	1509279300	1457995980	96.6	99.03	96.78
7	10617674	10617674	1592651100	1538413704	96.59	98.96	96.53
8	8572602	8572602	1285890300	1252982198	97.44	98.84	96.11

Sample	Raw Reads Number	Clean Reads Number	Raw Bases(bp)	Clean Bases(bp)	Effective Rate(%)	Q20 (%)	Q30 (%)
9	12247218	12247218	1837082700	1773272410	96.53	99.05	96.84
10	14081126	14081126	2112168900	2050203374	97.07	98.96	96.54
11	12505816	12505816	1875872400	1826674932	97.38	98.96	96.51
12	12680534	12680534	1902080100	1849925258	97.26	98.88	96.29
13	11683008	11683008	1752451200	1703880290	97.23	98.95	96.47
14	10564288	10564288	1584643200	1539260336	97.14	98.94	96.45
15	11492212	11492210	1723831800	1663929104	96.53	98.98	96.62
16	10433998	10433998	1565099700	1524064042	97.38	98.93	96.41
17	13606542	13606542	2040981300	1968553242	96.45	99.08	96.96
18	10592282	10592280	1588842300	1536830820	96.73	98.96	96.51
19	13545596	13545596	2031839400	1961355940	96.53	99.03	96.78
20	12062032	12062032	1809304800	1755352734	97.02	98.92	96.4
21	10927288	10927288	1639093200	1582337902	96.54	99.05	96.83
22	14367062	14367062	2155059300	2084840266	96.74	99	96.65
23	12867004	12867004	1930050600	1865652798	96.66	99.04	96.74
24	13234534	13234534	1985180100	1925959568	97.02	98.98	96.59
25	11318000	11318000	1697700000	1645978946	96.95	98.94	96.44
26	10295686	10295686	1544352900	1498509330	97.03	98.95	96.49
27	9459646	9459646	1418946900	1378947708	97.18	98.87	96.24
28	8931000	8931000	1339650000	1305473564	97.45	98.8	96
29	11171878	11171878	1675781700	1613286492	96.27	99.03	96.76
30	10514262	10514262	1577139300	1531467378	97.1	98.91	96.36
31	11765412	11765412	1764811800	1700634446	96.36	99.06	96.86
32	9707304	9707304	1456095600	1415509696	97.21	98.82	96.05
33	10811066	10811066	1621659900	1567852002	96.68	99.03	96.74
34	12958716	12958714	1943807400	1876882836	96.56	99.03	96.74
35	11996920	11996920	1799538000	1740537516	96.72	99.04	96.78
36	8238886	8238886	1235832900	1205947472	97.58	98.87	96.22
37	10220578	10220578	1533086700	1490271970	97.21	98.85	96.15
38	8589546	8589546	1288431900	1247620058	96.83	98.94	96.46
39	11018756	11018756	1652813400	1603690670	97.03	98.92	96.39
40	12629180	12629180	1894377000	1835507976	96.89	98.97	96.6
41	8452538	8452538	1267880700	1232986820	97.25	98.86	96.17
42	10491198	10491198	1573679700	1528029794	97.1	98.92	96.39
43	9460270	9460270	1419040500	1374330386	96.85	98.88	96.25
44	9851066	9851066	1477659900	1432044200	96.91	98.87	96.22
45	7697910	7697910	1154686500	1118938774	96.9	98.87	96.22
46	7456864	7456864	1118529600	1085318508	97.03	98.79	95.96
47	10962320	10962320	1644348000	1589097034	96.64	99	96.65
48	11372100	11372100	1705815000	1656123808	97.09	98.96	96.52
49	14979710	14979710	2246956500	2157408612	96.01	99.15	97.19
50	12892316	12892316	1933847400	1849898344	95.66	99.2	97.39
53	11879096	11879096	1781864400	1702408930	95.54	99.12	97.09

Sample	Raw Reads Number	Clean Reads Number	Raw Bases(bp)	Clean Bases(bp)	Effective Rate(%)	Q20 (%)	Q30 (%)
54	12469894	12469894	1870484100	1792303758	95.82	99.16	97.23
55	12462636	12462636	1869395400	1785206074	95.5	99.18	97.3
56	11761536	11761536	1764230400	1682413226	95.36	99.13	97.13
57	11463260	11463260	1719489000	1651834896	96.07	99.14	97.17
58	14607516	14607516	2191127400	2099086056	95.8	99.17	97.29
59	13271394	13271394	1990709100	1906589382	95.77	99.2	97.37
60	13124128	13124126	1968619200	1893591386	96.19	99.14	97.18
61	12698512	12698512	1904776800	1830714274	96.11	99.13	97.1
62	12379190	12379190	1856878500	1779255186	95.82	99.2	97.38
63	11510736	11510736	1726610400	1675723294	97.05	98.89	96.29
64	9985164	9985164	1497774600	1452511274	96.98	98.89	96.28
65	11812216	11812216	1771832400	1696790954	95.76	99.18	97.29
66	11692296	11692296	1753844400	1672814592	95.38	99.19	97.31
67	9361176	9361174	1404176400	1362036468	97	98.88	96.26
68	10611854	10611854	1591778100	1543924634	96.99	98.9	96.32
69	9033684	9033684	1355052600	1292900092	95.41	99.19	97.32
70	15393860	15393860	2309079000	2201278834	95.33	99.18	97.34
71	11437586	11437586	1715637900	1671479726	97.43	98.93	96.41
72	11057318	11057318	1658597700	1616357558	97.45	98.91	96.38
73	12444496	12444496	1866674400	1816711008	97.32	98.94	96.46
74	10172510	10172510	1525876500	1481866772	97.12	98.94	96.45
75	10530204	10530204	1579530600	1530611786	96.9	98.9	96.29
76	10932096	10932096	1639814400	1598234810	97.46	98.88	96.26
77	9916160	9916160	1487424000	1443828548	97.07	98.93	96.43
78	9769534	9769534	1465430100	1425406084	97.27	98.92	96.38
79	10070242	10070242	1510536300	1464314498	96.94	98.96	96.53
80	9655216	9655216	1448282400	1410177440	97.37	98.82	96.06
81	10703630	10703630	1605544500	1560576558	97.2	98.97	96.55
82	12913198	12913198	1936979700	1877901816	96.95	98.96	96.54
83	10852972	10852972	1627945800	1583250906	97.25	98.98	96.58
84	5997996	5997996	899699400	880173882	97.83	98.75	95.8
85	7916714	7916714	1187507100	1162650936	97.91	98.7	95.62
86	8657774	8657774	1298666100	1265004536	97.41	98.87	96.23
87	6278100	6278100	941715000	920222750	97.72	98.72	95.67
88	10481954	10481954	1572293100	1534806500	97.62	98.79	95.96
89	10311046	10311044	1546656900	1511494158	97.73	98.79	95.93
90	9116788	9116788	1367518200	1334782020	97.61	98.81	96.02
91	8231460	8231460	1234719000	1205329986	97.62	98.72	95.7
92	9877828	9877828	1481674200	1443675314	97.44	98.83	96.05
93	9375938	9375938	1406390700	1370986674	97.48	98.84	96.1
94	10721848	10721848	1608277200	1560129164	97.01	98.94	95.55
95	15679384	15679384	2351907600	2262681906	96.21	99.11	97.06
96	15292808	15292806	2293921200	2193731008	95.63	99.16	97.24

Sample	Raw Reads Number	Clean Reads Number	Raw Bases(bp)	Clean Bases(bp)	Effective Rate(%)	Q20 (%)	Q30 (%)
97	15477960	15477960	2321694000	2189916712	94.32	99.29	97.71
98	15590416	15590416	2338562400	2239732170	95.77	99.17	97.27
99	13420400	13420400	2013060000	1900971532	94.43	99.27	97.64
100	14517722	14517722	2177658300	2058829352	94.54	99.27	97.61
103	14630986	14630986	2194647900	2059832958	93.86	99.3	97.72
104	16148646	16148646	2422296900	2291000128	94.58	99.27	97.63
105	16752394	16752394	2512859100	2389310526	95.08	99.21	97.41
106	16710550	16710550	2506582500	2392567628	95.45	99.2	97.42
107	15605130	15605130	2340769500	2244742070	95.9	99.1	97.02
108	13125026	13125026	1968753900	1857084292	94.33	99.28	97.66
109	14027166	14027164	2104074900	1992586928	94.7	99.28	97.63
110	16398836	16398836	2459825400	2337346824	95.02	99.19	97.34
111	13565008	13565008	2034751200	1928970484	94.8	99.15	97.2
112	16376768	16376768	2456515200	2317390940	94.34	99.31	97.75
113	16116840	16116840	2417526000	2291460962	94.79	99.27	97.6
114	12241344	12241344	1836201600	1713379288	93.31	99.39	98.01
115	14276080	14276080	2141412000	2040861440	95.3	99.2	97.36
116	16692064	16692064	2503809600	2354275840	94.03	99.32	97.76
117	14689090	14689090	2203363500	2115658964	96.02	99.11	97.04
118	14962612	14962612	2244391800	2121833004	94.54	99.22	97.47
119	13620378	13620378	2043056700	1965640686	96.21	99.05	96.84
120	10564210	10564208	1584631500	1487492464	93.87	99.31	97.76
121	14132110	14132110	2119816500	2043330084	96.39	99.09	96.99
122	14533156	14533156	2179973400	2069985272	94.95	99.17	97.28
123	12906576	12906576	1935986400	1826475928	94.34	99.23	97.49
124	13561286	13561286	2034192900	1927746394	94.77	99.27	97.6
125	15258730	15258730	2288809500	2199784892	96.11	99.11	97.06
126	14556790	14556790	2183518500	2052107808	93.98	99.33	97.8
127	12597696	12597696	1889654400	1806011586	95.57	99.16	97.19
128	15588864	15588864	2338329600	2230082856	95.37	99.21	97.41
129	10887784	10887784	1633167600	1586141408	97.12	98.93	96.42
130	16787318	16787318	2518097700	2432172772	96.59	99.04	96.84
131	11310092	11310092	1696513800	1646916190	97.08	98.91	96.37
132	12618272	12618272	1892740800	1795264012	94.85	99.22	97.45
133	16118414	16118414	2417762100	2336391736	96.63	99.06	96.9
134	13654636	13654636	2048195400	1961075206	95.75	99.18	97.32
135	13192194	13192194	1978829100	1906690778	96.35	99.07	96.91
136	11959844	11959844	1793976600	1734030142	96.66	99.02	96.72
137	12304892	12304892	1845733800	1784925532	96.71	98.97	96.57
138	10679824	10679824	1601973600	1520046010	94.89	99.24	97.52
139	10084386	10084386	1512657900	1445338534	95.55	99.13	97.14
140	14146902	14146902	2122035300	2024955034	95.43	99.19	97.36
141	13337098	13337098	2000564700	1934316370	96.69	99.02	96.72

Sample	Raw Reads Number	Clean Reads Number	Raw Bases(bp)	Clean Bases(bp)	Effective Rate(%)	Q20 (%)	Q30 (%)
142	17245882	17245882	2586882300	2449547358	94.69	99.24	97.54
143	15834372	15834372	2375155800	2244805244	94.51	99.27	97.63
144	14763908	14763908	2214586200	2065862584	93.28	99.36	97.96
145	10760556	10760556	1614083400	1485881846	92.06	99.42	98.16
146	13703594	13703594	2055539100	1880679128	91.49	99.39	98.03
147	12095640	12095640	1814346000	1693057354	93.32	99.34	97.86
148	14065432	14065432	2109814800	1969324184	93.34	99.36	97.93
149	15038012	15038012	2255701800	2102895694	93.23	99.35	97.87
150	10968742	10968742	1645311300	1527260354	92.83	99.4	98.06
151	13450546	13450546	2017581900	1859639020	92.17	99.4	98.1
152	15725282	15725282	2358792300	2202889922	93.39	99.34	97.88
153	15617710	15617710	2342656500	2193743528	93.64	99.34	97.84
154	14971696	14971696	2245754400	2088104324	92.98	99.36	97.95
155	17001688	17001688	2550253200	2407943278	94.42	99.3	97.75
156	16137966	16137966	2420694900	2322075946	95.93	99.15	97.18
157	15995644	15995644	2399346600	2295593490	95.68	99.15	97.2
158	14512112	14512112	2176816800	2008317852	92.26	99.39	98.07
159	11231548	11231548	1684732200	1546996456	91.82	99.4	98.04
160	14895012	14895012	2234251800	2134483972	95.53	99.19	97.36
161	16254626	16254626	2438193900	2327198820	95.45	99.18	97.3
162	10944290	10944290	1641643500	1525148156	92.9	99.36	97.94
163	13572610	13572610	2035891500	1890067276	92.84	99.36	97.96
164	14439378	14439378	2165906700	2074202210	95.77	99.14	97.17
165	14430860	14430860	2164629000	2077219920	95.96	99.13	97.13
166	14826226	14826226	2223933900	2158120842	97.04	98.96	96.56
167	12985560	12985560	1947834000	1882326294	96.64	98.97	96.56
168	13841264	13841264	2076189600	1985393108	95.63	99.16	97.2
169	14442238	14442238	2166335700	2106493940	97.24	98.93	96.44
170	13499230	13499230	2024884500	1958422978	96.72	98.97	96.57
171	14480266	14480266	2172039900	2080767010	95.8	99.15	97.21
172	13414542	13414542	2012181300	1940256336	96.43	99.09	96.99
173	12407674	12407674	1861151100	1801832334	96.81	99	96.66
174	13491724	13491724	2023758600	1959427484	96.82	99.01	96.7
175	12513124	12513124	1876968600	1812465918	96.56	98.98	96.61
176	13769674	13769674	2065451100	1999358380	96.8	98.98	96.61
177	11273014	11273014	1690952100	1646694696	97.38	98.85	96.15
178	12771678	12771678	1915751700	1866336962	97.42	98.8	96
179	10922984	10922984	1638447600	1594851420	97.34	98.85	96.14
180	7901254	7901254	1185188100	1153596934	97.33	98.86	96.17
181	14863066	14863066	2229459900	2160373184	96.9	98.99	96.68
182	11908700	11908700	1786305000	1737548686	97.27	98.89	96.29
183	10591824	10591824	1588773600	1543100608	97.13	98.89	96.31
184	13169088	13169088	1975363200	1914134366	96.9	99	96.65

Sample	Raw Reads Number	Clean Reads Number	Raw Bases(bp)	Clean Bases(bp)	Effective Rate(%)	Q20 (%)	Q30 (%)
185	12164382	12164382	1824657300	1773661348	97.21	98.9	96.28
186	10479162	10479162	1571874300	1520814182	96.75	99.02	96.73
187	8401064	8401064	1260159600	1223560576	97.1	98.78	95.91
188	11240858	11240856	1686128700	1628385480	96.58	99.01	96.68
189	10024136	10024136	1503620400	1450994542	96.5	98.98	96.62
190	10107102	10107102	1516065300	1465432710	96.66	98.96	96.5
191	9794926	9794926	1469238900	1425895208	97.05	98.91	96.37
192	9379680	9379680	1406952000	1368703516	97.28	98.86	96.15
193	10410374	10410374	1561556100	1512007874	96.83	99	96.69
194	9476478	9476478	1421471700	1379752654	97.07	98.92	96.39
195	8030978	8030978	1204646700	1170592194	97.17	98.82	96.06
196	10584542	10584542	1587681300	1533375020	96.58	98.99	96.63
197	8350040	8350040	1252506000	1221022378	97.49	98.87	96.22
198	10244714	10244714	1536707100	1493616610	97.2	98.83	96.07
199	9233914	9233914	1385087100	1343916780	97.03	98.85	96.08
200	9241652	9241652	1386247800	1348008740	97.24	98.85	96.13
201	10303950	10303950	1545592500	1481647570	95.86	99.09	96.97
202	7663708	7663708	1149556200	1117721972	97.23	98.82	96.04
203	10658760	10658760	1598814000	1549627254	96.92	98.93	96.41
204	9250892	9250892	1387633800	1351087814	97.37	98.87	96.2
205	8997518	8997518	1349627700	1315061080	97.44	98.87	96.19
206	9934724	9934724	1490208600	1450137318	97.31	98.83	96.06
207	10164214	10164214	1524632100	1482983288	97.27	98.87	96.25
208	12981924	12981924	1947288600	1874606980	96.27	99.09	96.99
209	8636706	8636706	1295505900	1257240842	97.05	98.89	96.31
210	9494262	9494262	1424139300	1384565140	97.22	98.8	95.96
211	10570584	10570584	1585587600	1539477128	97.09	98.88	96.24
212	10817730	10817730	1622659500	1574000896	97	98.93	96.43
213	9665556	9665556	1449833400	1401526150	96.67	99.01	96.74
214	8473908	8473908	1271086200	1237574238	97.36	98.82	96.04
215	10138606	10138606	1520790900	1473514466	96.89	98.95	96.49
216	11104300	11104300	1665645000	1619358618	97.22	98.96	95.64
217	8925178	8925178	1338776700	1293299758	96.6	98.98	96.6
218	7346914	7346914	1102037100	1069894466	97.08	98.77	95.87
219	9311486	9311486	1396722900	1357025636	97.16	98.86	96.21
220	10471024	10471024	1570653600	1519136816	96.72	99	96.66
221	8589160	8589160	1288374000	1252960056	97.25	98.86	96.2
222	9327164	9327164	1399074600	1359067018	97.14	98.82	96.04
223	7672932	7672932	1150939800	1121682656	97.46	98.73	95.74
224	10131532	10131532	1519729800	1470814792	96.78	98.93	96.4
225	7615512	7615512	1142326800	1105376226	96.77	98.83	96.07
226	8409444	8409444	1261416600	1218503768	96.6	98.88	96.22
227	8100138	8100138	1215020700	1178124658	96.96	98.83	96.08

Sample	Raw Reads Number	Clean Reads Number	Raw Bases(bp)	Clean Bases(bp)	Effective Rate(%)	Q20 (%)	Q30 (%)
228	7942382	7942382	1191357300	1160839600	97.44	98.81	95.99
229	9728652	9728652	1459297800	1411801742	96.75	99.01	96.71
230	9202006	9202006	1380300900	1340592036	97.12	98.78	95.89
231	7006762	7006762	1051014300	1022104352	97.25	98.78	95.91
232	8830644	8830644	1324596600	1291751278	97.52	98.76	95.81
233	7054222	7054222	1058133300	1030685118	97.41	98.75	95.81
234	7503734	7503734	1125560100	1094846410	97.27	98.72	95.68
235	10607530	10607530	1591129500	1537649382	96.64	98.94	96.46
236	9833186	9833186	1474977900	1421905160	96.4	99.03	96.77
237	11733304	11733304	1759995600	1696632844	96.4	99.09	97.02
238	12703192	12703192	1905478800	1814409210	95.22	99.18	97.28
239	10829838	10829838	1624475700	1557218958	95.86	99.1	97.02
240	9730976	9730976	1459646400	1410463938	96.63	99.02	96.72
241	10916378	10916376	1637456700	1569866620	95.87	99.12	97.09
242	9726188	9726188	1458928200	1405162230	96.31	99.03	96.76
243	11059592	11059592	1658938800	1592940840	96.02	99.06	96.9
244	11690384	11690384	1753557600	1683043790	95.98	99.11	97.07
245	8806112	8806112	1320916800	1275345968	96.55	99.07	96.9
246	10859602	10859602	1628940300	1567269756	96.21	99.03	96.77
247	9794362	9794362	1469154300	1417502550	96.48	98.97	96.56
248	12941190	12941190	1941178500	1859342082	95.78	99.07	96.91
249	6034646	6034646	905196900	878970838	97.1	98.84	96.12
250	9542664	9542664	1431399600	1388402894	97	98.88	96.22
251	11606522	11606522	1740978300	1669619886	95.9	99.11	97.08
252	10495756	10495752	1574363400	1520001028	96.55	99.09	97
253	8672872	8672872	1300930800	1262208148	97.02	98.9	96.34
254	7708374	7708374	1156256100	1131164098	97.83	98.73	95.72
255	10770658	10770658	1615598700	1559639950	96.54	98.97	96.6
256	11806472	11806472	1770970800	1697869610	95.87	99.12	97.08
257	7933696	7933696	1190054400	1159623112	97.44	98.82	96.04
258	7845234	7845234	1176785100	1147912098	97.55	98.74	95.78
259	9135308	9135308	1370296200	1336225088	97.51	98.78	95.9
260	9960422	9960422	1494063300	1446412624	96.81	98.89	96.25
261	8167400	8167400	1225110000	1182984818	96.56	98.95	96.48
262	7542152	7542152	1131322800	1102827886	97.48	98.78	95.88
263	8324462	8324462	1248669300	1218466142	97.58	98.78	95.87
264	6480952	6480952	972142800	946678374	97.38	98.79	95.93
265	9181456	9181456	1377218400	1335005526	96.93	98.94	96.47
266	7507096	7507096	1126064400	1096767870	97.4	98.72	95.67
267	6394616	6394616	959192400	929431078	96.9	98.8	95.98
268	7928324	7928324	1189248600	1157149356	97.3	98.81	96.01
269	7255430	7255430	1088314500	1061801738	97.56	98.78	95.92
270	7681456	7681456	1152218400	1125716816	97.7	98.66	95.48

Sample	Raw Reads Number	Clean Reads Number	Raw Bases(bp)	Clean Bases(bp)	Effective Rate(%)	Q20 (%)	Q30 (%)
271	5589216	5589216	838382400	815282672	97.24	98.64	95.41
272	9157336	9157336	1373600400	1335318286	97.21	98.83	96.07
273	8187872	8187872	1228180800	1197906206	97.54	98.82	96.03
274	7455052	7455052	1118257800	1085875892	97.1	98.8	95.94
275	8564636	8564636	1284695400	1246015380	96.99	98.87	96.22
276	6321914	6321914	948287100	930004790	98.07	98.66	95.48
277	8196692	8196690	1229503800	1198091428	97.45	98.79	95.92
278	7453688	7453688	1118053200	1089953948	97.49	98.69	95.57
279	6804740	6804740	1020711000	992010980	97.19	98.66	95.5
280	8824654	8824654	1323698100	1278139996	96.56	98.87	95.26
281	10093950	10093950	1514092500	1468183312	96.97	98.92	95.42
282	10998968	10998968	1649845200	1585937694	96.13	99.01	95.84

Notes:

Sample: Sample name;Raw Reads Number: Number of reads in the sample before quality control;

Clean Reads Number: Number of reads in the sample after quality control;

Raw Bases (bp): Total number of bases in the sample before quality control, calculated as the product of the number of sequencing reads and the length of sequencing reads, in base pairs (bp);

Clean Bases (bp): Total number of bases in the sample after quality control, equal to the sum of the lengths of clean reads, in base pairs (bp);

Effective Rate (%): Ratio of the total number of bases in the sample after quality control to that before quality control;

Q20, Q30: Ratios of the number of bases with quality scores greater than 20 and 30, respectively, to the total number of bases in the sample after quality control.

Table S3. Quality statistics of sequencing data

Sample	NA_number	NA_rate (%)	Het_alt_number	Het_alt_rate (%)	Hom_alt_number	Hom_alt_rate (%)	Ref_number	Ref_rate(%)
1	1033	1.94	369	0.71	19487	37.38	32273	61.91
2	668	1.26	331	0.63	16085	30.64	36078	68.73
3	937	1.76	2189	4.19	15617	29.9	34419	65.91
4	827	1.56	546	1.04	16624	31.76	35165	67.19
5	1035	1.95	554	1.06	18137	34.79	33436	64.14
6	1231	2.32	301	0.58	17907	34.48	33723	64.94
7	932	1.75	1187	2.27	17389	33.29	33654	64.43
8	1024	1.93	1666	3.2	13917	26.69	36555	70.11
9	392	0.74	584	1.11	15407	29.2	36779	69.7
10	529	1	169	0.32	12235	23.25	40229	76.43
11	435	0.82	380	0.72	14299	27.12	38048	72.16
12	788	1.48	342	0.65	19436	37.11	32596	62.24
13	622	1.17	539	1.03	15523	29.55	36478	69.43
14	834	1.57	572	1.09	18213	34.81	33543	64.1
15	477	0.9	224	0.43	14778	28.05	37683	71.53
16	698	1.31	345	0.66	11385	21.7	40734	77.64
17	388	0.73	400	0.76	15540	29.45	36834	69.8
18	670	1.26	725	1.38	10400	19.81	41367	78.81
19	316	0.59	174	0.33	16104	30.47	36568	69.2
20	704	1.32	631	1.2	15542	29.63	36285	69.17
21	647	1.22	270	0.51	17445	33.22	34800	66.27
22	391	0.74	227	0.43	18624	35.29	33920	64.28
23	414	0.78	223	0.42	18610	35.28	33915	64.3
24	343	0.65	241	0.46	16363	30.98	36215	68.56
25	665	1.25	1542	2.94	13260	25.26	37695	71.8
26	672	1.26	306	0.58	13643	25.99	38541	73.43
27	652	1.23	429	0.82	13685	26.06	38396	73.12
28	940	1.77	801	1.53	13515	25.88	37906	72.59
29	593	1.12	395	0.75	13921	26.48	38253	72.77
30	632	1.19	691	1.32	13593	25.88	38246	72.81
31	546	1.03	216	0.41	16790	31.91	35610	67.68
32	988	1.86	279	0.53	16948	32.48	34947	66.98
33	730	1.37	301	0.57	16025	30.56	36106	68.86
34	582	1.09	354	0.67	15848	30.14	36378	69.19
35	774	1.46	295	0.56	19223	36.69	32870	62.74
36	691	1.3	753	1.44	16494	31.43	35224	67.13
37	704	1.32	134	0.26	10807	20.6	41517	79.14
38	737	1.39	269	0.51	11165	21.3	40991	78.19
39	607	1.14	2443	4.65	11193	21.3	38919	74.05
40	389	0.73	1309	2.48	10920	20.69	40544	76.83
41	869	1.63	292	0.56	15278	29.22	36723	70.23
42	843	1.59	4564	8.72	13488	25.78	34267	65.5

Sample	NA_number	NA_rate (%)	Het_alt_number	Het_alt_rate (%)	Hom_alt_number	Hom_alt_rate (%)	Ref_number	Ref_rate(%)
43	770	1.45	458	0.87	16830	32.12	35104	67
44	777	1.46	1361	2.6	16431	31.37	34593	66.04
45	1023	1.92	3797	7.28	14250	27.33	34092	65.39
46	1043	1.96	1297	2.49	15709	30.14	35113	67.37
47	466	0.88	2033	3.86	16247	30.83	34416	65.31
48	502	0.94	1380	2.62	16436	31.21	34844	66.17
49	336	0.63	822	1.56	9529	18.04	42475	80.41
50	582	1.09	1301	2.47	16520	31.42	34759	66.11
53	483	0.91	820	1.56	14277	27.1	37582	71.34
54	538	1.01	394	0.75	18268	34.71	33962	64.54
55	368	0.69	450	0.85	16569	31.38	35775	67.76
56	404	0.76	933	1.77	15224	28.86	36601	69.38
57	369	0.69	278	0.53	16455	31.17	36060	68.3
58	402	0.76	2393	4.54	12928	24.5	37439	70.96
59	415	0.78	850	1.61	13760	26.09	38137	72.3
60	508	0.96	2547	4.84	11428	21.7	38679	73.46
61	523	0.98	668	1.27	12879	24.47	39092	74.26
62	462	0.87	1898	3.6	13082	24.82	37720	71.57
63	613	1.15	789	1.5	14444	27.49	37316	71.01
64	831	1.56	905	1.73	14803	28.29	36623	69.98
65	618	1.16	2842	5.41	14142	26.91	35560	67.68
66	617	1.16	584	1.11	14859	28.28	37102	70.61
67	807	1.52	422	0.81	14914	28.49	37019	70.71
68	477	0.9	339	0.64	16318	30.97	36028	68.38
69	475	0.89	518	0.98	15577	29.57	36592	69.45
70	269	0.51	1739	3.29	15268	28.87	35886	67.85
71	453	0.85	612	1.16	15658	29.71	36439	69.13
72	536	1.01	993	1.89	15877	30.17	35756	67.94
73	579	1.09	349	0.66	16195	30.8	36039	68.54
74	462	0.87	324	0.61	14991	28.45	37385	70.94
75	471	0.89	1149	2.18	14955	28.38	36587	69.44
76	531	1	324	0.62	14849	28.21	37458	71.17
77	596	1.12	673	1.28	16299	31.01	35594	67.71
78	626	1.18	1254	2.39	17246	32.83	34036	64.79
79	423	0.8	168	0.32	15038	28.51	37533	71.17
80	850	1.6	303	0.58	18374	35.12	33635	64.3
81	573	1.08	660	1.26	12008	22.83	39921	75.91
82	421	0.79	3405	6.46	11496	21.8	37840	71.75
83	546	1.03	5446	10.35	11245	21.37	35925	68.28
84	2105	3.96	294	0.58	17385	34.05	33378	65.37
85	1352	2.54	313	0.6	17598	33.97	33899	65.43
86	776	1.46	1300	2.48	16341	31.19	34745	66.32

Sample	NA_number	NA_rate (%)	Het_alt_number	Het_alt_rate (%)	Hom_alt_number	Hom_alt_rate (%)	Ref_number	Ref_rate(%)
87	1366	2.57	1355	2.62	13891	26.82	36550	70.57
88	615	1.16	213	0.41	18516	35.24	33818	64.36
89	607	1.14	210	0.4	18548	35.29	33797	64.31
90	1054	1.98	265	0.51	17824	34.21	34019	65.29
91	1158	2.18	273	0.52	17752	34.14	33979	65.34
92	573	1.08	217	0.41	16267	30.93	36105	68.66
93	645	1.21	208	0.4	16249	30.94	36060	68.66
94	900	1.69	5122	9.8	15451	29.56	31689	60.63
95	284	0.53	243	0.46	18650	35.27	33985	64.27
96	184	0.35	136	0.26	12324	23.26	40518	76.48
97	283	0.53	240	0.45	18650	35.27	33989	64.28
98	494	0.93	236	0.45	15085	28.64	37347	70.91
99	249	0.47	1173	2.22	15220	28.76	36520	69.02
100	295	0.55	243	0.46	18639	35.26	33985	64.28
103	317	0.6	301	0.57	11279	21.34	41265	78.09
104	467	0.88	214	0.41	11127	21.12	41354	78.48
105	201	0.38	259	0.49	16393	30.95	36309	68.56
106	258	0.49	750	1.42	16768	31.7	35386	66.89
107	225	0.42	245	0.46	16388	30.96	36304	68.58
108	626	1.18	306	0.58	18036	34.33	34194	65.09
109	239	0.45	249	0.47	16378	30.95	36296	68.58
110	266	0.5	1341	2.54	13805	26.1	37750	71.37
111	403	0.76	309	0.59	13398	25.39	39052	74.02
112	198	0.37	211	0.4	11902	22.47	40851	77.13
113	319	0.6	1909	3.61	16228	30.71	34706	65.68
114	635	1.19	345	0.66	18619	35.45	33563	63.9
115	310	0.58	842	1.59	17891	33.85	34119	64.56
116	423	0.8	297	0.56	16260	30.83	36182	68.61
117	194	0.36	428	0.81	9935	18.76	42605	80.44
118	346	0.65	1066	2.02	18256	34.57	33494	63.42
119	380	0.71	2000	3.79	15914	30.15	34868	66.06
120	396	0.74	892	1.69	17825	33.78	34049	64.53
121	415	0.78	2081	3.95	17080	32.38	33586	63.67
122	350	0.66	271	0.51	13600	25.75	38941	73.74
123	252	0.47	173	0.33	15198	28.72	37539	70.95
124	316	0.59	1336	2.53	15966	30.21	35544	67.26
125	183	0.34	233	0.44	12266	23.15	40480	76.41
126	434	0.82	387	0.73	16466	31.23	35875	68.04
127	417	0.78	992	1.88	16521	31.32	35232	66.8
128	513	0.96	291	0.55	15058	28.6	37300	70.85
129	485	0.91	672	1.28	18322	34.78	33683	63.94
130	218	0.41	199	0.38	11899	22.47	40846	77.15

Sample	NA_number	NA_rate (%)	Het_alt_number	Het_alt_rate (%)	Hom_alt_number	Hom_alt_rate (%)	Ref_number	Ref_rate(%)
131	504	0.95	498	0.95	13710	26.04	38450	73.02
132	644	1.21	348	0.66	18603	35.42	33567	63.92
133	186	0.35	145	0.27	12218	23.06	40613	76.66
134	288	0.54	284	0.54	18580	35.14	34010	64.32
135	476	0.9	275	0.52	16250	30.84	36161	68.63
136	586	1.1	274	0.52	17225	32.76	35077	66.72
137	312	0.59	742	1.4	16688	31.58	35420	67.02
138	503	0.95	648	1.23	11287	21.43	40724	77.34
139	604	1.14	182	0.35	11060	21.04	41316	78.61
140	369	0.69	2405	4.56	12287	23.27	38101	72.17
141	490	0.92	481	0.91	18915	35.91	33276	63.18
142	457	0.86	439	0.83	18996	36.04	33270	63.12
143	578	1.09	318	0.6	18065	34.35	34201	65.04
144	495	0.93	392	0.74	14956	28.4	37319	70.86
145	607	1.14	297	0.57	15185	28.89	37073	70.54
146	588	1.11	296	0.56	15215	28.94	37063	70.5
147	552	1.04	266	0.51	14994	28.5	37350	70.99
148	539	1.01	417	0.79	15094	28.68	37112	70.52
149	542	1.02	494	0.94	15118	28.73	37008	70.33
150	584	1.1	515	0.98	15186	28.88	36877	70.14
151	655	1.23	391	0.74	19649	37.42	32467	61.83
152	348	0.65	375	0.71	16210	30.69	36229	68.6
153	280	0.53	479	0.91	15069	28.5	37334	70.6
154	301	0.57	3041	5.75	13739	25.99	36081	68.26
155	387	0.73	605	1.15	14243	26.99	37927	71.87
156	207	0.39	426	0.8	16650	31.44	35879	67.75
157	296	0.56	598	1.13	18028	34.1	34240	64.77
158	636	1.2	800	1.52	19299	36.74	32427	61.74
159	498	0.94	981	1.86	16373	31.09	35310	67.05
160	497	0.93	675	1.28	17634	33.48	34356	65.23
161	467	0.88	696	1.32	16966	32.2	35033	66.48
162	458	0.86	1209	2.29	13381	25.39	38114	72.32
163	552	1.04	477	0.91	14958	28.43	37175	70.66
164	327	0.62	1184	2.24	13892	26.29	37759	71.47
165	427	0.8	210	0.4	16842	31.94	35683	67.66
166	229	0.43	1343	2.54	15183	28.68	36407	68.78
167	650	1.22	292	0.56	17084	32.53	35136	66.91
168	224	0.42	553	1.04	11174	21.11	41211	77.85
169	257	0.48	550	1.04	9990	18.88	42365	80.08
170	450	0.85	1195	2.27	17849	33.86	33668	63.87
171	344	0.65	1325	2.51	16471	31.18	35022	66.31
172	276	0.52	976	1.85	16946	32.04	34964	66.11

Sample	NA_number	NA_rate (%)	Het_alt_number	Het_alt_rate (%)	Hom_alt_number	Hom_alt_rate (%)	Ref_number	Ref_rate(%)
173	576	1.08	332	0.63	15024	28.57	37230	70.8
174	505	0.95	856	1.63	14783	28.07	37018	70.3
175	615	1.16	379	0.72	15280	29.08	36888	70.2
176	547	1.03	744	1.41	14601	27.75	37270	70.84
177	689	1.3	556	1.06	14982	28.55	36935	70.39
178	614	1.15	658	1.25	14840	28.24	37050	70.51
179	571	1.07	2282	4.34	11643	22.14	38666	73.52
180	949	1.79	337	0.65	14585	27.93	37291	71.42
181	217	0.41	976	1.84	15014	28.36	36955	69.8
182	399	0.75	218	0.41	15915	30.16	36630	69.42
183	598	1.12	728	1.38	16866	32.09	34970	66.53
184	328	0.62	284	0.54	13209	25	39341	74.46
185	619	1.16	264	0.5	14840	28.24	37439	71.25
186	390	0.73	174	0.33	15525	29.42	37073	70.25
187	1124	2.11	175	0.34	15076	28.97	36787	70.69
188	744	1.4	274	0.52	11192	21.35	40952	78.13
189	885	1.66	220	0.42	16477	31.52	35580	68.06
190	798	1.5	198	0.38	15033	28.71	37133	70.91
191	811	1.53	1182	2.26	11087	21.18	40082	76.56
192	791	1.49	153	0.29	15571	29.73	36647	69.98
193	673	1.27	273	0.52	17361	33.08	34855	66.4
194	934	1.76	903	1.73	16623	31.83	34702	66.44
195	819	1.54	129	0.25	14644	27.98	37570	71.78
196	671	1.26	264	0.5	18269	34.8	33958	64.69
197	903	1.7	186	0.36	14875	28.46	37198	71.18
198	805	1.51	1226	2.34	16581	31.67	34550	65.99
199	1041	1.96	193	0.37	14789	28.37	37139	71.26
200	950	1.79	227	0.43	16035	30.71	35950	68.85
201	1036	1.95	338	0.65	19493	37.4	32295	61.96
202	1724	3.24	381	0.74	15627	30.38	35430	68.88
203	1200	2.26	470	0.9	19036	36.63	32456	62.46
204	729	1.37	169	0.32	15597	29.75	36667	69.93
205	927	1.74	187	0.36	15541	29.75	36507	69.89
206	863	1.62	175	0.33	15122	28.91	37002	70.75
207	994	1.87	289	0.55	18656	35.76	33223	63.68
208	545	1.03	1093	2.08	15468	29.4	36056	68.53
209	859	1.62	138	0.26	12901	24.67	39264	75.07
210	1159	2.18	357	0.69	19304	37.12	32342	62.19
211	848	1.6	163	0.31	16217	31	35934	68.69
212	892	1.68	1052	2.01	14391	27.53	36827	70.46
213	740	1.39	3230	6.16	12081	23.05	37111	70.79
214	1410	2.65	883	1.71	17844	34.48	33025	63.81

Sample	NA_number	NA_rate (%)	Het_alt_number	Het_alt_rate (%)	Hom_alt_number	Hom_alt_rate (%)	Ref_number	Ref_rate(%)
215	581	1.09	105	0.2	10475	19.92	42001	79.88
216	504	0.95	177	0.34	10407	19.76	42074	79.9
217	740	1.39	117	0.22	10835	20.67	41470	79.11
218	1461	2.75	124	0.24	11250	21.76	40327	78
219	1132	2.13	632	1.21	13279	25.52	38119	73.26
220	766	1.44	333	0.64	13590	25.94	38473	73.43
221	889	1.67	1688	3.23	12945	24.76	37640	72.01
222	994	1.87	649	1.24	14571	27.93	36948	70.83
223	1780	3.35	298	0.58	14437	28.1	36647	71.32
224	903	1.7	164	0.31	15947	30.52	36148	69.17
225	1330	2.5	129	0.25	14381	27.75	37322	72.01
226	1310	2.46	175	0.34	15552	29.99	36125	69.67
227	1108	2.08	122	0.23	13807	26.52	38125	73.24
228	1379	2.59	165	0.32	16204	31.29	35414	68.39
229	848	1.6	148	0.28	15351	29.34	36815	70.37
230	1111	2.09	148	0.28	15998	30.74	35905	68.98
231	1767	3.32	185	0.36	14239	27.71	36971	71.94
232	973	1.83	185	0.35	18317	35.1	33687	64.55
233	1329	2.5	190	0.37	17727	34.2	33916	65.43
234	1409	2.65	828	1.6	13519	26.12	37406	72.28
235	1046	1.97	775	1.49	13416	25.74	37925	72.77
236	848	1.6	241	0.46	17186	32.85	34887	66.69
237	803	1.51	887	1.69	15203	29.04	36269	69.27
238	536	1.01	124	0.24	10817	20.55	41685	79.21
239	877	1.65	2237	4.28	15725	30.08	34323	65.65
240	747	1.41	443	0.85	11311	21.58	40661	77.58
241	795	1.5	203	0.39	14668	28.01	37496	71.6
242	1177	2.21	501	0.96	15287	29.41	36197	69.63
243	779	1.47	924	1.76	16406	31.32	35053	66.92
244	1312	2.47	338	0.65	17728	34.19	33784	65.16
245	1128	2.12	241	0.46	17114	32.89	34679	66.65
246	914	1.72	285	0.55	17840	34.14	34123	65.31
247	1298	2.44	269	0.52	17672	34.07	33923	65.41
248	454	0.85	243	0.46	16299	30.92	36166	68.62
249	2300	4.33	601	1.18	12461	24.5	37800	74.32
250	1182	2.22	2829	5.44	12521	24.09	36630	70.47
251	954	1.79	350	0.67	19538	37.42	32320	61.91
252	697	1.31	459	0.87	15938	30.38	36068	68.75
253	971	1.83	459	0.88	12992	24.89	38740	74.23
254	1343	2.53	302	0.58	10585	20.43	40932	78.99
255	662	1.25	299	0.57	17227	32.81	34974	66.62
256	732	1.38	599	1.14	10591	20.2	41240	78.66

Sample	NA_number	NA_rate (%)	Het_alt_number	Het_alt_rate (%)	Hom_alt_number	Hom_alt_rate (%)	Ref_number	Ref_rate(%)
257	1317	2.48	239	0.46	14959	28.85	36647	70.69
258	1394	2.62	278	0.54	10637	20.55	40853	78.92
259	1400	2.63	179	0.35	11577	22.37	40006	77.29
260	1062	2	314	0.6	18259	35.05	33527	64.35
261	1405	2.64	281	0.54	18103	34.98	33373	64.48
262	1863	3.5	169	0.33	13966	27.22	37164	72.45
263	1022	1.92	437	0.84	11337	21.74	40366	77.42
264	1931	3.63	271	0.53	16684	32.57	34276	66.9
265	874	1.64	475	0.91	15572	29.78	36241	69.31
266	1523	2.86	187	0.36	15834	30.66	35618	68.97
267	1955	3.68	411	0.8	11070	21.62	39726	77.58
268	1096	2.06	144	0.28	11370	21.84	40552	77.89
269	1614	3.04	2250	4.36	14672	28.46	34626	67.17
270	1486	2.8	539	1.04	14779	28.6	36358	70.36
271	3412	6.42	571	1.15	11559	23.23	37620	75.62
272	1522	2.86	3534	6.84	14591	28.26	33515	64.9
273	1352	2.54	520	1	11762	22.7	39528	76.29
274	3749	7.05	7224	14.62	6978	14.12	35211	71.26
275	1064	2	94	0.18	10318	19.8	41686	80.01
276	2359	4.44	1498	2.95	12865	25.32	36440	71.73
277	1327	2.5	250	0.48	17764	34.27	33821	65.25
278	2076	3.91	2625	5.14	13117	25.68	35344	69.19
279	3392	6.38	12039	24.19	8618	17.32	29113	58.5
280	746	1.4	210	0.4	15480	29.53	36726	70.07
281	455	0.86	137	0.26	14895	28.26	37675	71.48
282	746	1.4	428	0.82	15580	29.72	36408	69.46

Notes:

ID: Name of the SNP;Chrom: Name of the chromosome where the SNP is located;

Position: Chromosomal position of the SNP;

Ref: Genotype of this locus in the reference genome;

NA_rate: Missing rate of the current locus across all tested samples;

Ref_rate: Proportion of the non-mutant genotype at the current locus among all tested samples;

Hom_alt_rate: Proportion of the homozygous mutant genotype at the current locus among all tested samples;

Het_alt_rate: Proportion of the heterozygous mutant genotype at the current locus among all tested samples;

Table S4. SNP Variation Annotation Classification Comparison Table

Function_type	The number of SNPs	Proportion
UTR3	1694	3.17%
UTR5	953	1.78%
UTR5;UTR3	5	0.01%

Function_type	The number of SNPs	Proportion
downstream	1816	3.40%
exonic	23337	43.67%
exonic;splicing	2	0%
intergenic	16857	31.54%
intronic	5727	10.72%
ncRNA_exonic	522	0.98%
ncRNA_intronic	195	0.36%
ncRNA_splicing	2	0%
splicing	19	0.04%
upstream	2084	3.9%
upstream;downstream	228	0.43%

Notes:

UTR3: The variation is located in the 3' untranslated region (UTR) of the gene;

UTR5: The variation is located in the 5' untranslated region (UTR) of the gene;

UTR5;UTR3: The variation is located in both the 5' untranslated region (UTR) and the 3' untranslated region (UTR) of the gene;

downstream: The variation is located in the 2 Kbp downstream region of the gene;

exonic: The variation is located in the exonic coding region of the gene;

exonic;splicing: The variation is located in both the exonic coding region and the splice site (2 bp of the intron adjacent to the exon/intron boundary);

intergenic: The variation is located in the intergenic region;

intronic: The variation is located in the intronic region of the gene;

ncRNA_exonic: The variation is located in the exonic region of non-coding RNA (ncRNA);

ncRNA_intronic: The variation is located in the intronic region of non-coding RNA (ncRNA);

ncRNA_splicing: The variation is located in the splice site of non-coding RNA (ncRNA) (2 bp of the intron adjacent to the exon/intron boundary);

splicing: The variation is located in the splice site (2 bp of the intron adjacent to the exon/intron boundary);

upstream: The variation is located in the 2 Kbp upstream region of the gene;upstream;

downstream: The variation is located in both the 2 Kbp upstream region and the 2 Kbp downstream region of the gene.

Table S5. The statistical table of significant SNP loci identified in the GWAS analysis

Phenotype	Chr_SNP	P
NNASI	7_154995077_154995077	1.93E-05
	7_154998819_154998819	3.04E-05
	1_294735828_294735828	3.17E-05
	1_233913206_233913206	3.73E-05
NNED	1_194115137_194115137	9.39E-05
	1_195528622_195528622	7.32E-05
	1_26005278_26005278	1.23E-05

	2_23522183_23522183	1.66E-05
	2_238220502_238220502	9.30E-05
	2_24249673_24249673	3.35E-05
	7_115812783_115812783	9.68E-05
	2_199942104_199942104	1.36E-05
	5_10171673_10171673	6.65E-05
**NNEH**	2_199946413_199946413	6.70E-05
	7_160388139_160388139	8.37E-05
	2_34011937_34011937	8.39E-05
	1_72269916_72269916	6.41E-05
	2_226148184_226148184	1.74E-06
	2_238901644_238901644	9.11E-05
**NNEL**	4_6013389_6013389	5.16E-05
	4_6016367_6016367	4.99E-05
	6_115081967_115081967	2.59E-06
	2_258649_258649	1.41E-05
	2_194915314_194915314	2.43E-05
**NNHKW**	1_20538910_20538910	2.72E-05
	5_39508218_39508218	6.19E-05
	1_42126116_42126116	7.56E-05
	1_25117152_25117152	5.20E-05
	1_25803354_25803354	1.94E-05
	1_26042527_26042527	1.29E-05
	1_27820667_27820667	9.12E-05
	1_27829120_27829120	1.69E-05
	1_27835262_27835262	1.49E-06
	1_28086366_28086366	8.11E-06
	1_28483137_28483137	1.56E-05
**NNKNR**	1_28569039_28569039	1.73E-05
	1_28578589_28578589	3.13E-06
	1_29958397_29958397	1.45E-05
	1_72244851_72244851	7.24E-05
	1_72269916_72269916	7.06E-06
	2_145686003_145686003	8.69E-05
	2_236374525_236374525	2.20E-05
	2_238901644_238901644	9.48E-05
	1_181154957_181154957	8.82E-05
	2_199942104_199942104	3.97E-05
	2_199946413_199946413	4.01E-05
**NNPH**	2_201908097_201908097	6.58E-06
	2_202498149_202498149	7.33E-06
	2_202673370_202673370	1.00E-04
	2_203789058_203789058	6.14E-05
	2_204487841_204487841	2.20E-05

	2_209839830_209839830	8.31E-06
	2_210086505_210086505	8.64E-06
	2_210092307_210092307	2.92E-05
	2_210101937_210101937	5.09E-05
	2_210163111_210163111	8.67E-05
NNKRN	4_203405970_203405970	2.56797E-05
	10_87129259_87129259	3.54207E-05
	4_204261479_204261479	4.82577E-05
	6_9907408_9907408	6.66241E-05
NNSPAD	4_6139554_6139554	1.1708E-05
	8_165308041_165308041	2.14846E-05
NNGY	1_194115137_194115137	3.45E-05
	1_194693323_194693323	2.05E-05
	1_195528622_195528622	4.96E-05
	1_25117152_25117152	3.13E-05
	1_26042527_26042527	1.00E-04
	1_26185422_26185422	6.54E-05
	1_27835262_27835262	7.24E-05
	1_28086366_28086366	1.54E-05
	1_28578589_28578589	7.96E-05
	1_5435310_5435310	9.89E-05
	4_207459424_207459424	1.01E-05
	4_207466246_207466246	6.66E-06
LNED	1_194115137_194115137	8.71E-05
	1_272124012_272124012	5.66E-05
	1_272131030_272131030	5.66E-05
	1_273843805_273843805	2.58E-05
	1_282304076_282304076	5.15E-05
	1_44536041_44536041	2.21E-05
	2_110346030_110346030	8.45E-06
	2_110794735_110794735	8.01E-06
	2_128509430_128509430	9.20E-06
	2_241898187_241898187	1.17E-05
	2_241935696_241935696	6.34E-06
	2_3408060_3408060	3.72E-05
	2_70946481_70946481	3.53E-05
	3_151841150_151841150	9.69E-05
	5_54725264_54725264	2.73E-05
	5_54727804_54727804	2.73E-05
	6_160174143_160174143	2.58E-07
	7_102366740_102366740	3.69E-06
	7_115743986_115743986	4.64E-05
	7_115812783_115812783	3.94E-06
	7_115913001_115913001	8.54E-06

	9_128356218_128356218	5.22E-05
	9_21987134_21987134	2.91E-05
	9_25068825_25068825	1.01E-05
	9_31420482_31420482	1.31E-05
	9_31555548_31555548	6.99E-05
	2_210163111_210163111	1.88E-05
**LNEH**	2_210175234_210175234	7.92E-05
	2_199942104_199942104	8.29E-05
	2_238901644_238901644	8.70E-05
	6_115081967_115081967	5.71E-05
	6_149171788_149171788	8.78E-05
**LNEL**	6_151448184_151448184	7.15E-05
	8_120538965_120538965	9.48E-05
	8_120553121_120553121	3.38E-05
	8_120714204_120714204	6.33E-05
	1_18770933_18770933	5.66E-05
	1_19005849_19005849	2.11E-05
	1_29126030_29126030	1.81E-05
**LNHKW**	10_147850207_147850207	5.84E-05
	10_148009352_148009352	1.64E-05
	8_1107019_1107019	3.07E-05
	7_146763671_146763671	3.07E-06
**LNKNR**	7_155262483_155262483	5.90E-06
	5_199614683_199614683	5.76E-05
	2_241935696_241935696	5.00E-06
	3_197655490_197655490	1.96E-05
**LNKRN**	5_177627248_177627248	3.34E-05
	5_177539875_177539875	7.35E-05
	1_280161904_280161904	1.34413E-07
**LNSPAD**	1_281620027_281620027	6.10945E-05
	5_223397191_223397191	6.46741E-05
	1_46377097_46377097	8.09363E-05
	1_194115137_194115137	1.36E-05
	1_194693323_194693323	8.12E-05
**LNGY**	2_3408060_3408060	1.85E-05
	7_146763671_146763671	5.82E-05
	1_16415334_16415334	7.39E-05
	1_16415338_16415338	7.39E-05
	1_16439127_16439127	5.18E-05
**LNASI**	1_20264204_20264204	4.29E-06
	1_20264352_20264352	4.29E-06
	1_20264376_20264376	4.29E-06
	1_20264616_20264616	2.78E-06
	1_250104887_250104887	8.56E-05

	10_116186478_116186478	7.62E-05
	9_74468497_74468497	9.13E-05
	9_80227437_80227437	7.93E-05
	9_80354029_80354029	2.47E-05
	9_80902913_80902913	7.26E-05
	9_81462991_81462991	2.73E-05
	9_81842105_81842105	8.44E-05
	2_202498149_202498149	8.73098E-05
	2_209839830_209839830	1.49075E-05
	2_210086505_210086505	1.33752E-05
**LNPH**	2_210163111_210163111	7.65609E-06
	2_210175234_210175234	5.08215E-06
	5_218593404_218593404	3.80479E-05
	8_159803727_159803727	4.32466E-05

Table S6. Names of the experimental inbred line materials and low nitrogen tolerance index.

Number/Sample	Inbred line name	Average low nitrogen tolerance index
1	Chang7-2	0.96
2	Zheng58	1.08
3	W1	1.08
4	W2	1.03
5	W3	1.03
6	W4	0.86
7	W5	1.03
8	W6	1.00
9	Shi1447	1.00
10	Shi516	1.01
11	M41	0.97
12	Shi779	1.04
13	Shi259	0.97
14	Shi58	0.93
15	Shi2791	0.99
16	HAN-9	1.00
17	22N187	1.03
18	HAN38	1.03
19	JMC01	0.96
20	Shiqing344	1.00
21	21Q151	1.03

22	23N3	0.98
23	23N4	0.99
24	23N6	1.02
25	21n403	1.09
26	21N409	0.94
27	21N416	1.06
28	21N417	0.99
29	21N418	0.89
30	21N420	0.95
31	HF1383	1.02
32	23N2	0.89
33	X14	1.00
34	X128	1.11
35	13N201	0.97
36	HAN8-1	1.00
37	HAN8-2	0.97
38	HAN10-1	1.18
39	HAN15	1.02
40	HAN41	1.09
41	HAN60	1.03
42	HAN99	0.98
43	HAN1055	1.14
44	HAN1056	0.99
45	HAN106	0.96
46	HAN109	0.93
47	HAN110	0.90
48	HAN1131	1.03
49	HAN136	0.93
50	Xindan65	1.01
51	Huawan617	1.00
52	23N36	1.05
53	23N37	1.03
54	23N38	1.05
55	23N39	0.96
56	23N40	1.04
57	23N41	1.14
58	23N42	0.98
59	23N43	1.04
60	23N44	0.98
61	23N45	1.00
62	23N46	0.92
63	23N48	1.02
64	HAN12	0.99
65	HAN23	0.99

66	HAN76	1.05
67	Jia1fu	0.88
68	x132	0.94
69	HAN113	1.00
70	Shi359*Shi516	0.96
71	Shiz528*W25	1.02
72	NF171*W25	1.04
73	23N60	0.98
74	23N61	0.92
75	23N62	0.98
76	JQ9603-2	0.91
77	23N64	0.92
78	23N65	0.94
79	23N66	0.96
80	21Q112	0.95
81	21Q15	0.92
82	21n404	0.98
83	x129	1.03
84	702F	0.95
85	335F	1.03
86	x129-2	1.00
87	L335F	1.06
88	z528*W25-1	0.99
89	S528*W25-2	1.04
90	21Q28	0.97
91	HAN10	0.95
92	HAN371	0.90
93	23N87	1.02
94	HAN1071	0.95
95	23N89	0.98
96	P3	1.00
97	23N91	0.91
98	NF173*W25	1.01
99	NGF23011	1.08
100	HAN1051	0.95
101	NGF23052	0.91
102	H1712CV	0.97
103	NGF23071	1.03
104	HAN50	1.00
105	XY1665	0.98
106	YD9953	1.02
107	H1710CV	1.09
108	HAN1072	1.11

109	HAN130	1.04
110	XD65	1.00
111	ZM-1	0.97
112	NGF2309	1.03
113	23N108	0.93
114	23N109	0.96
115	NF172	0.98
116	HAN2A	0.93
117	HAN1052	0.98
118	23N115	1.07
119	H1713CV	1.05
120	NGF2301	0.84
121	NGF2302	0.97
122	NGF2305	0.95
123	NF17	1.12
124	ph4CV	0.96
125	NGF2307	0.91
126	NGF2308	1.12
127	HAN8A	0.95
128	HAN36	0.97
129	HAN37	1.08
130	HAN78	1.04
131	HAN111	1.03
132	HAN126	1.03
133	HAN133	0.97
134	HAN135	0.89
135	LP3	0.99
136	23N136	1.10
137	23N137	0.96
138	23N138	0.99
139	23N139	1.07
140	23N140	0.96
141	23N142	0.91
142	23N143	0.96
143	23N144	1.04
144	23N145	1.04
145	23N146	1.00
146	23N147	0.97
147	H1710PHCV	1.03
148	YH-3	1.03
149	HAN2C	1.01
150	HAN29	1.03
151	23N153	0.93
152	23N154	1.13

153	23N155	1.09
154	23N156	1.06
155	HF138-1	1.01
156	23N158	0.91
157	M39-2*S1447	0.94
158	23N160	1.08
159	23N161	0.98
160	HAN43	1.06
161	HAN49	1.03
162	HAN93	0.94
163	HAN105	0.89
164	HAN107	1.04
165	Shi88	0.98
166	HAN2	1.04
167	23N173	0.92
168	23N174	0.94
169	23N175	1.01
170	23N176	1.01
171	23N177	0.95
172	23N178	1.08
173	NF17*W25	1.01
174	W25	1.01
175	M51	0.94
176	H14	1.05
177	23N184	1.03
178	Shi153	1.01
179	6WC*W25	0.96
180	W25-3	1.05
181	VK22-4-1	1.14
182	23N190	0.97
183	23N192	0.90
184	HAN8C	0.93
185	L91158	1.02
186	L1173	0.95
187	DK517	1.01
188	HAN381	1.02
189	L117	1.10
190	J42	1.01
191	J2416K	1.08
192	S359	0.94
193	S153	1.00
194	Z495	1.01
195	Z598	1.00
196	J421	0.97

197	23N210	0.93
198	23N211	0.90
199	23N220	0.99
200	23N234	0.94
201	23N239	0.94
202	23N242	0.97
203	23N244	0.94
204	23N245	0.96
205	23N246	0.97
206	23N247	0.97
207	23N248	1.02
208	23N250	1.04
209	23N251	0.96
210	23N252	0.95
211	23N253	1.00
212	23N254	0.94
213	23N255	1.13
214	23N256	0.94
215	23N257	0.99
216	23N258	0.91
217	23N259	1.00
218	23N260	0.97
219	23N261	0.98
220	23N262	1.00
221	23N263	1.02
222	23N264	0.94
223	23N265	0.90
224	23N266	1.03
225	23N267	0.96
226	23N268	0.99
227	23N269	0.98
228	23N270	1.00
229	23N271	1.02
230	23N272	1.21
231	23N273	0.99
232	23N274	1.12
233	23N275	0.98
234	23N276	0.97
235	23N277	1.01
236	23N278	1.04
237	23N279	0.87
238	23N280	0.90
239	23N281	0.98
240	23N282	0.99

241	23N283	1.02
242	23N284	1.08
243	23N285	0.99
244	23N287	0.86
245	23N288	1.02
246	23N290	0.90
247	23N291	1.14
248	23N292	0.96
249	23N293	0.98
250	23N294	1.00
251	23N295	0.94
252	H1710	1.20
253	23N297	0.95
254	23N298	0.96
255	23N299	0.99
256	23N300	0.93
257	2023NY4	0.96
258	2023NY8	1.02
259	2023NY9	0.99
260	2023NY14	0.90
261	2023NY17	0.96
262	2023NY27	0.89
263	2023NY28	0.95
264	2023NY29	1.05
265	2023NY30	0.98
266	2023NY31	1.07
267	2023NY32	0.96
268	2023NY33	0.95
269	2023NY35	1.02
270	2023NY38	1.03
271	2023NY40	0.96
272	2023NY41	1.03
273	2023NY42	0.98
274	2023NY43	0.96
275	2023NY44	1.03
276	2023NY46	0.94
277	2023NY47	0.99
278	2023NY48	0.95
279	2023NY49	0.93
280	2023NY50	0.97
281	2023NY51	0.98
282	2023NY52	0.96

Figure S1. Statistical Chart of Daily Mean Temperature and Precipitation at the Experimental Site in 2024 and 2025

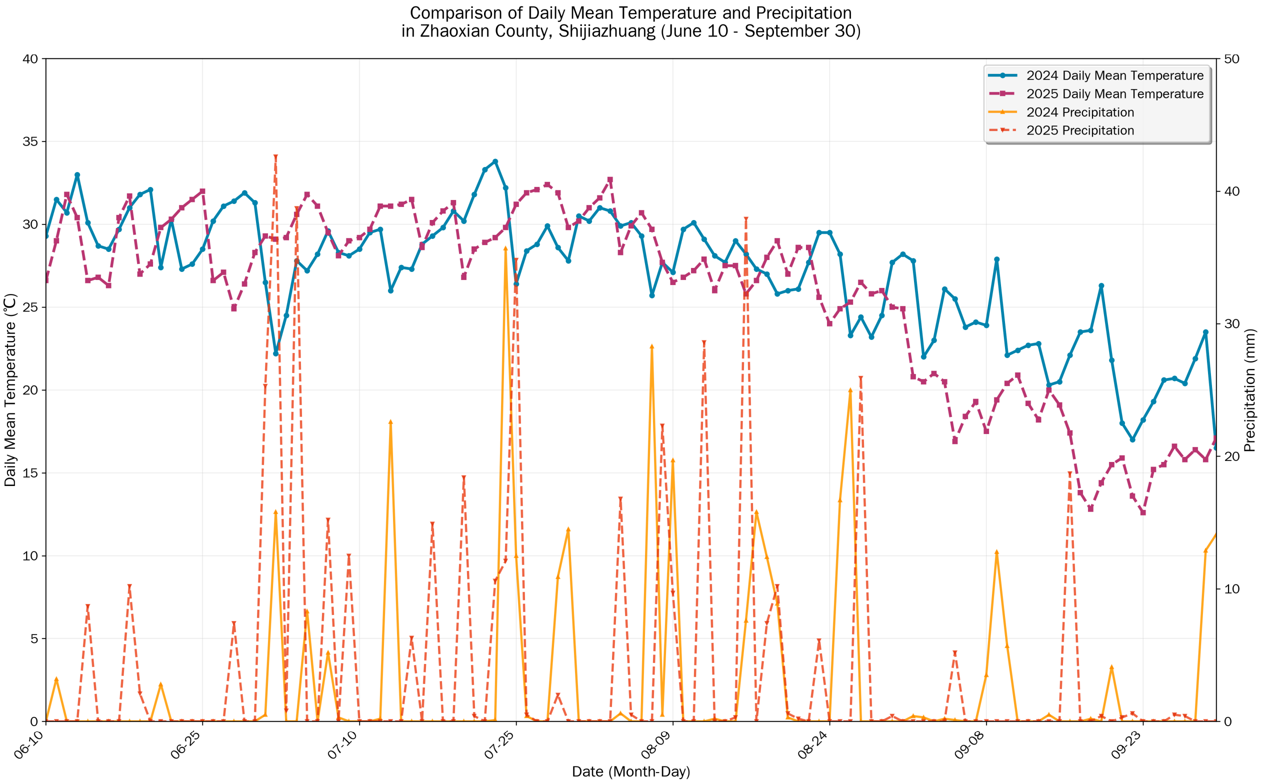

Figure S2. Comparison result statistics chart

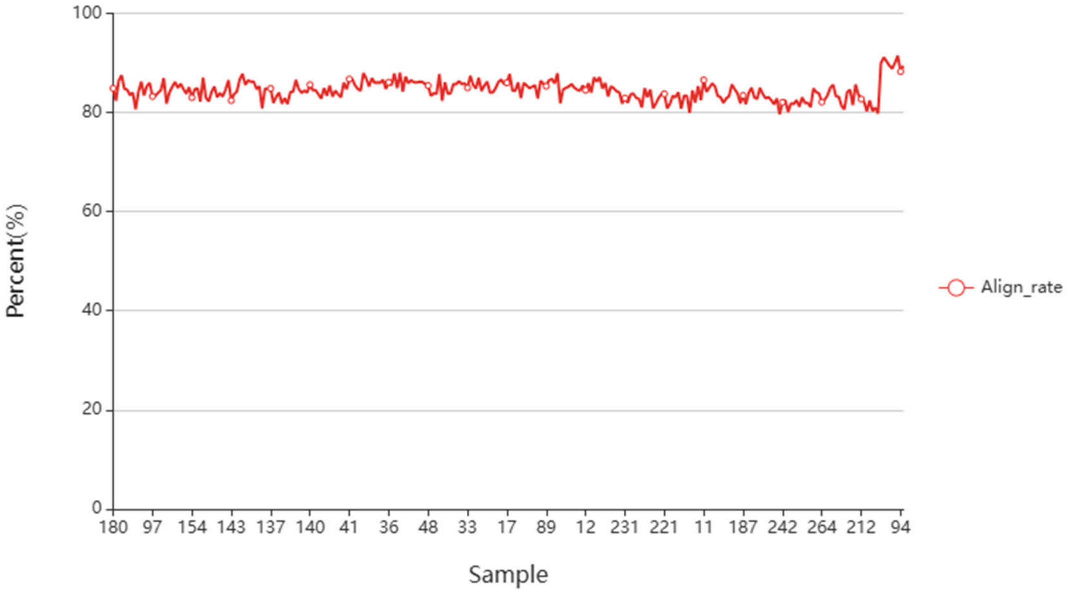

Note: The higher the Align_rate, the higher the similarity between the sample sequencing data and the reference genome.

Figure S3. Core SNP Loci Statistical Chart

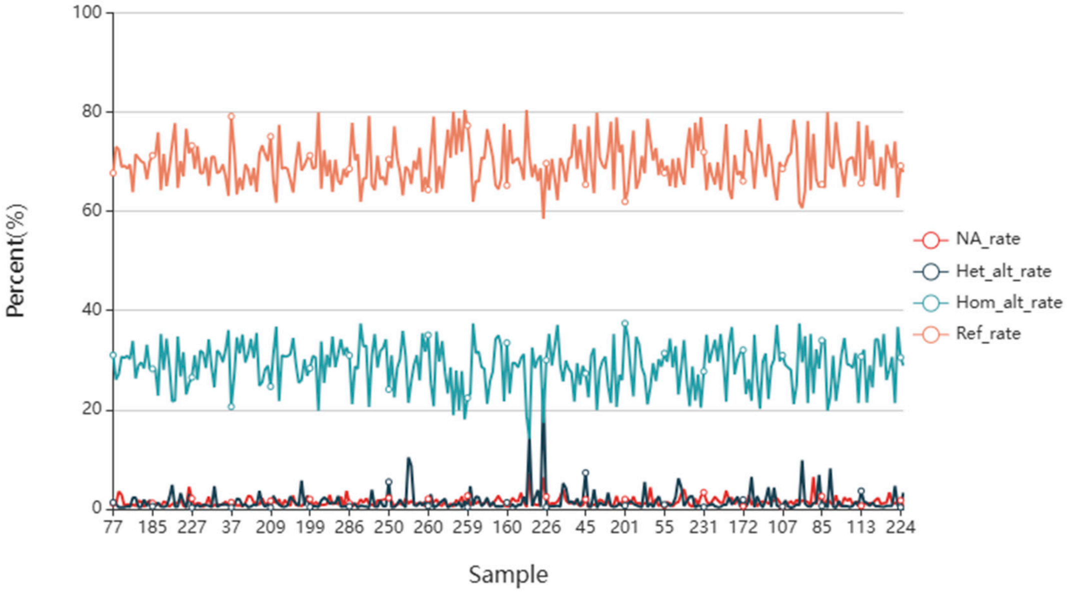

Note: The abscissa represents sample names, and the ordinate represents percentage. An NA_rate ⩽ 10% indicates that the sample detection missing rate meets the required standard; the lower the NA_rate, the fewer the number of undetected loci in the sample.

Sliding window analysis was performed across chromosomes with a window size of 10 kb to count the number of SNPs within each window. The genomic distribution of core SNP loci is shown in the following figure:

Figure S4. Distribution Map of Core SNP Loci

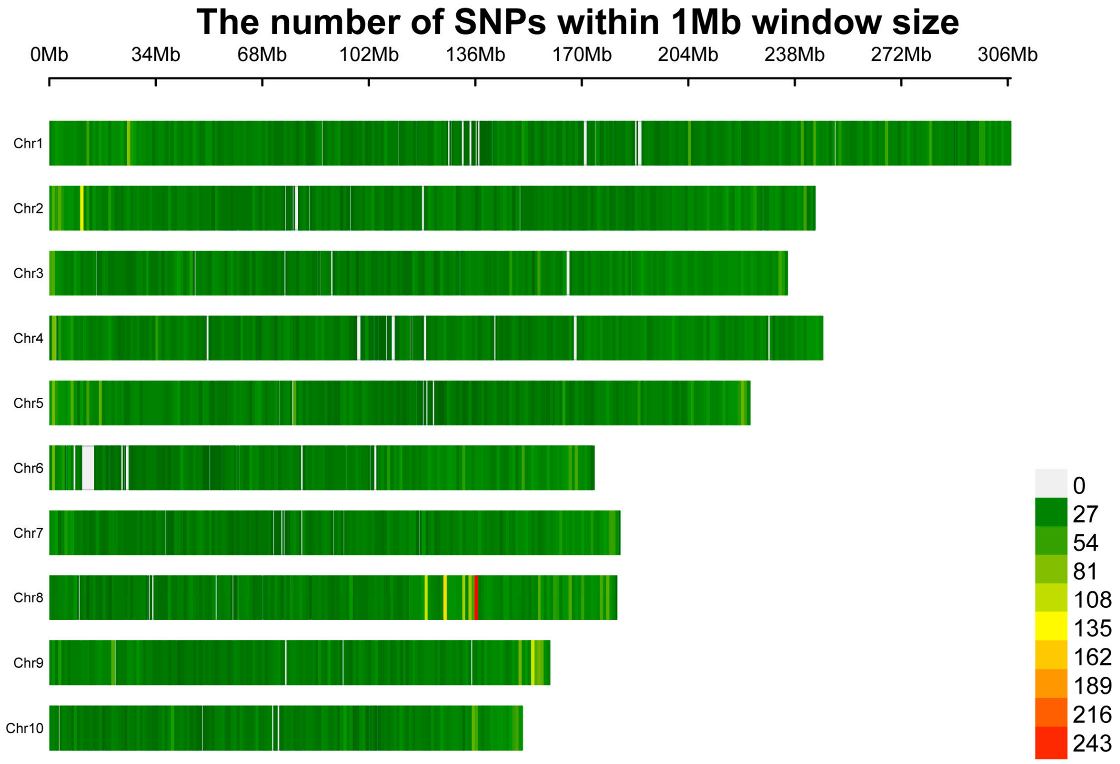

Note: The numbers 1 through 10 on the left represent the chromosomes
